# Stratified Causal Inference for Intensive Care Unit Risk Prediction: Informatics-Based Modeling of Anesthetic Drug Combinations

**DOI:** 10.2196/80294

**Published:** 2026-02-25

**Authors:** Junqi Cui, Weijia Li, Enoch Chi Ngai Lim, Xiaoqin Wu, Chi Eung Danforn Lim

**Affiliations:** 1 School of Health Sciences, University of New South Wales, Kensington, Australia; 2 Dongfang Hospital, Beijing University of Chinese Medicine, Beijing, China; 3 Translational Research Department, Specialist Medical Services Group, Earlwood, Australia; 4 Sydney Institute of Traditional Chinese Medicine, Haymarket, Australia; 5 NICM Health Research Institute, Western Sydney University, 158 Hawkesbury Rd, Westmead, 2145, Australia, 61 295547788; 6 School of Life Sciences, University of Technology Sydney, Ultimo, Australia

**Keywords:** fentanyl, propofol, intensive care, dose-response analysis, counterfactual modeling

## Abstract

**Background:**

Postoperative intensive care unit (ICU) admission affects 15% to 20% of surgical patients and represents a major source of morbidity and health care costs. Current anesthetic dosing relies on empirical guidelines rather than individualized risk assessment. We developed a counterfactual dose-response model to identify optimal fentanyl-propofol combinations.

**Objective:**

This study aimed to develop and evaluate a stratified, causal machine learning framework using electronic health record data to identify optimal fentanyl-propofol dose combinations and predict postoperative ICU admission risk, enabling precision anesthesia and individualized clinical decision support.

**Methods:**

We analyzed perioperative electronic health records of 67,134 surgical procedures from UC Irvine Medical Center (2017‐2022). A hierarchical learning framework was used to estimate causal effects while controlling for confounding variables. A total of 6 dose-sensitive subgroups were identified through stratified analysis. The primary end point was postoperative ICU admission.

**Results:**

High-risk combinations (fentanyl >5 mcg/kg with propofol <1 mg/kg) increased ICU admissions’ absolute risk difference by 36% (absolute risk increase; 95% CI 0.351-0.509; *P*<.001). A total of 6 patient subgroups demonstrated distinct dose-response patterns, with populations considered vulnerable (high glucose, elevated creatinine) showing elevated risk even at standard doses. The optimal dose range for decision-making was determined to be 1.25 to 4.25 mg/kg for propofol and 3.5 to 4.0 mcg/kg for fentanyl.

**Conclusions:**

Fentanyl-propofol combinations exhibit complex, nonlinear dose-response relationships with ICU admission risk. High-dose combinations markedly increase risk through synergistic effects, while specific patient subgroups require enhanced monitoring even at standard doses. These findings support the development of individualized dosing algorithms and risk assessment tools that could inform future decision support tools aimed at reducing postoperative ICU use, although their predictive performance and clinical impact would require external validation.

## Introduction

Postoperative intensive care unit (ICU) admission represents a serious complication of surgical care and, according to global data, contributes substantially to postoperative mortality, highlighting significant gaps in critical care access and delivery [1]. Despite advances in perioperative monitoring and anesthetic techniques, current approaches to drug dosing rely primarily on empirical guidelines and clinical experience rather than individualized risk assessment based on patient characteristics and drug interactions [2,3].

The combination of fentanyl and propofol, used in millions of surgical procedures worldwide, exemplifies this challenge. While both agents are essential components of modern anesthesia, their complex pharmacological interactions and dose-dependent effects on postoperative outcomes remain poorly characterized [4,5]. Current dosing guidelines provide broad ranges without specific guidance for individual patient risk factors or drug interaction effects, potentially exposing patients considered vulnerable to preventable complications requiring intensive care support [2].

Fentanyl is a potent synthetic opioid that can enhance the hypnotic effect of propofol, thereby effectively reducing the dosage of propofol and further improving the hemodynamic stability of patients [4,6]. Fentanyl and propofol demonstrate synergistic effects that can reduce individual drug requirements while maintaining effective anesthesia [4,5]. However, high-dose combinations may exacerbate cardiovascular and respiratory depression through additive mechanisms, potentially leading to hemodynamic instability and respiratory failure requiring ICU support [1,7].

Postoperative admission to the ICU represents one of the most resource-intensive outcomes. Treatment-resistant hypotension or respiratory failure is a common cause of ICU transfer [8-10]. An increasing number of studies emphasize the need for more precise intraoperative anesthesia management [2,3,10]. The implementation of an accurate medication strategy is anticipated to effectively decrease the risk of postoperative ICU admission [2,3]. Recent large-scale perioperative studies and safety reviews [1,10] have reinforced concerns regarding opioid-related respiratory depression, hemodynamic instability, and unplanned ICU admission following general anesthesia, particularly in heterogeneous surgical populations.

Causal machine learning, by estimating individualized treatment effects, is reshaping how medical evidence is generated and translated into patient-level decision-making [11,12]. Doubly robust orthogonal estimators integrate learning algorithms with principled confounding adjustment in high-dimensional observational data, thereby enhancing the validity of causal inference [13].

Recent clinical applications include causal forests combined with double machine learning estimation of average and heterogeneous effects of tuberculosis preventive therapy on antiretroviral therapy adherence [14] and meta-learning analyses of midwife-led continuity of care on low birth weight with individualized treatment effect recovery [15].

Building on this foundation, we developed a comprehensive counterfactual dose-response modeling framework to characterize both the joint dosing effects and treatment effect heterogeneity of fentanyl-propofol coadministration, enabling the identification of dose combinations associated with more favorable postoperative risk profiles and the delineation of high-risk patient subgroups to support individualized anesthesia management and reduce ICU-related risks.

## Methods

### Study Design

We performed a retrospective analysis of perioperative electronic health records for 67,134 surgical procedures at the University of California, Irvine Medical Center (2017‐2022) [16]. This large, single-center cohort provides robust statistical power for detecting clinically meaningful dose-response relationships while maintaining consistent practice patterns and data quality standards. We excluded surgeries with an anesthesia duration of less than 60 minutes, patients younger than 18 years, patients aged 85 years or older, and cases lacking preoperative metabolic assessments. This study used fully deidentified electronic health records with no direct patient contact or intervention.

### Data Preprocessing

We included preoperative patient data available within 7 days prior to surgery, reflecting real-world clinical decision-making factors, including demographic characteristics (eg, age and sex), comorbidities (eg, hypertension, diabetes mellitus, hyperlipidemia, and chronic kidney disease), surgical characteristics (eg, procedure category), physiological parameters (eg, blood pressure, oxygen saturation, American Society of Anesthesiologists physical status, and pain score), and laboratory values (eg, complete blood count and comprehensive metabolic panel).

Fentanyl and propofol were administered intravenously via a peripheral vein from anesthesia induction until the end of surgery. For each patient, the total cumulative intraoperative dose was derived by summing all bolus administrations and the total amount delivered via continuous infusion over the intraoperative period. Doses were subsequently weight standardized by dividing the cumulative dose by body weight (eg, fentanyl [mcg/kg] and propofol [mg/kg]). Postoperative ICU admission was defined as ICU admission occurring within 24 hours after the end of surgery.

Variables with a missing value ratio exceeding 20% in either dataset were excluded from the analysis. For the remaining variables with missing values, multiple imputation using chained equations [17] was performed independently within each cohort. This approach addresses a critical methodological concern: because multiple imputations using chained equations rely on the mean and SD of the observed data for each variable, performing imputation separately for the 2 cohorts is essential to minimize bias and preserve the internal validity of the imputed values within each group. A schematic representation of the temporal and causal structure of covariates, intraoperative dosing, and postoperative ICU outcome is provided in Figure S5 in Multimedia Appendix 1.

### Covariate Classification

The treatment variable was defined as a 2D vector comprising intraoperative fentanyl dose and propofol dose. The outcome variable was postoperative ICU admission, coded as a binary variable. We categorized patient characteristics based on their likelihood of receiving certain drug combinations (propensity score) and their expected response to treatment (average treatment effect). All the selected confounding factors satisfy the backdoor criterion [18].

We evaluated 9 machine learning models for constructing the adjustment set and automatically tuned the hyperparameters for each model. Light gradient boosting machine was identified as the optimal model for constructing the adjustment set. After adjustment, the standardized mean difference (SMD) for most confounding factors was below the conventional threshold of 0.1, with a mean SMD of 0.0405. Multicollinearity was evaluated by calculating the variance inflation factor of the model design matrix. Variables with an SMD0.10 or a variance inflation factor greater than 5 were excluded.

### Total Eligible Cohort Analysis

We used the CausalForestDML model to calculate conditional average treatment effects. This approach identifies which patients are most likely to benefit from or be harmed by specific dose combinations, supporting personalized anesthetic management. On the basis of the conditional average treatment effects estimates, we constructed a causal decision tree (CDT) to identify potential effect modifiers. Each CDT was constrained to a maximum depth of 6. This restriction reflects a fundamental principle of hierarchical learning and represents the upper bound of model complexity permitted within our framework. We selected 6 subgroups defined by the terminal nodes of the CDT for heterogeneity treatment effect analysis.

### Dual-Exposure Cohort Analysis

We developed an extreme gradient boosting–based risk surface model to estimate group-level postoperative ICU admission risks associated with varying intraoperative fentanyl and propofol dose combinations in the dual-exposure cohort. We constructed a logistic regression model incorporating interaction terms to evaluate whether there was a statistically significant interaction between fentanyl and propofol doses.

To minimize the potential bias introduced by treatment effect heterogeneity, we identified 6 groups that were highly sensitive to combined medication dosages through heterogeneity treatment effect analysis. For each group, we independently trained XGBoost models with consistent hyperparameters.

### Interpretation Framework

We used the explainable boosting machine [19] model to quantitatively evaluate the contribution of each predictor variable to the risk of ICU admission. We trained XGBoost models across 6 sensitive groups to elucidate the observed heterogeneity in treatment responses, computing the Shapley additive explanation (SHAP) values of the primary explanatory variables.

### Ethical Considerations

This study was exempt from ethics review because it involved the analysis of publicly available, deidentified data. No human participants were directly involved, and no identifiable personal information was accessed.

This study was conducted using retrospective, fully deidentified patient data extracted from a single-center database. All datasets used were previously approved for research and made available through the Medical Informatics Operating Room Vitals and Events Repository (MOVER) data portal. The MOVER dataset was created under institutional review board approval at the University of California, Irvine, with a waiver of individual informed consent because all protected health information was removed or deidentified in a Health Insurance Portability and Accountability Act–compliant manner. Access to MOVER was obtained after application and execution of the University of California, Irvine, Operating Room Data Usage Agreement, which prohibits reidentification attempts and redistribution of the data and requires users to notify the dataset administrators if any potentially identifying information is detected. As all datasets were deidentified and released for secondary research use, the study was exempt from additional institutional review board approval, and informed consent was not required. All analyses complied with the ethical standards of the Declaration of Helsinki and relevant national regulations regarding secondary use of deidentified health data. Formal documentation of the institutional ethics exemption or waiver has been obtained and will be provided upon request.

## Results

### Cohort

**Figure 1.**
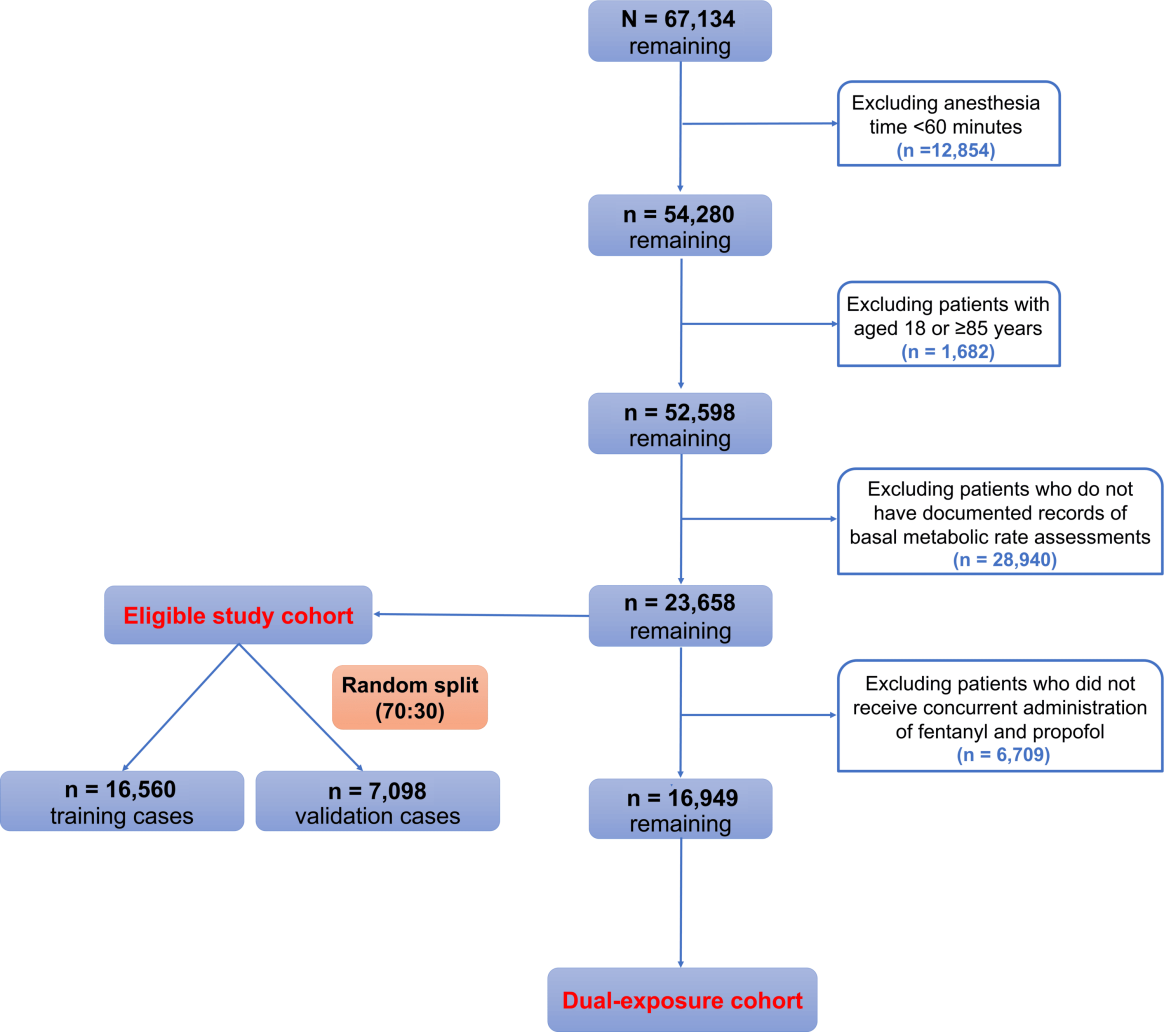
Study flowchart.

As illustrated in Figure 1, a total of 23,658 surgical cases were included in the analysis cohort, with 16,949 (71.6%) cases receiving both fentanyl and propofol (dual-exposure cohort) used for primary dose-response modeling.

Binary variables are presented as n (%), and continuous variables are presented as median (IQR). The postoperative ICU outcome rate of the total eligible cohort was 66.64%. The postoperative ICU outcome rate of the dual-exposure cohort was 64.26%.

As presented in Table 1, baseline characteristics revealed clinically substantial patterns associated with ICU admission risk. Patients requiring ICU admission were more likely to be men (9979, 63.3% vs 4347, 55.1%), older (median 55, IQR 39-66 years vs median 50, IQR 35-64 years), and had higher ASA physical status than those not requiring ICU admission. Laboratory parameters showed concerning patterns in ICU patients with lower hemoglobin (g/dL) (median 11.09, IQR 9.14-12.80 vs median 12.10, IQR 11.30-13.70), higher glucose (mg/dL) (median 130, IQR 108.33-163 vs median 109, IQR 96-132), and elevated creatinine. Notably, patients admitted to the ICU postoperatively received higher fentanyl doses (median 1.60, IQR 1.10-2.52 mcg/kg vs median 1.47, IQR 1.08-2.14 mcg/kg) but similar propofol doses (median 2.00, IQR 1.47-2.56 mg/kg vs median 2.13, 1.72-2.59 mg/kg).

**Table 1.** Baseline characteristics of the interpolated cohorts.

Variable	Total eligible cohort^a^			Dual-exposure cohort		
	ICU^b^ admission (no)	ICU admission (yes)	*P* value	ICU admission (no)	ICU admission (yes)	*P* value
Demographics						
Age (years), median (IQR)	50 (35-64)	55 (39-66)	<.001	47 (33-61)	54 (38-65)	<.001
Sex, n (%)			<.001			<.001
Male	4347 (55.1)	9979 (63.3)		3321 (54.8)	6806 (62.5)	
Female	3543 (44.9)	5789 (36.7)		2737 (45.2)	4085 (37.5)	
Body weight (kg), median (IQR)	76.50 (64.70-90.67)	75.90 (63.79-89.90)	.08	77.11 (65.16-90.72)	76.25 (64.00-90.23)	.007
ASA^c^ physical status, n (%)			<.001			<.001
1	705 (8.9)	328 (2.1)		649 (10.7)	304 (2.8)	
2	2892 (36.7)	2417 (15.3)		2607 (43.0)	2120 (19.5)	
3	3832 (48.6)	8524 (54.1)		2601 (42.9)	6340 (58.2)	
4	440 (5.6)	3996 (25.3)		200 (3.3)	2025 (18.6)	
5	21 (0.3)	503 (3.2)		1 (0.0)	102 (0.9)	
Pain score, n (%)			<.001			<.001
0	1981 (25.1)	3856 (24.5)		1115 (18.4)	2543 (23.3)	
1	609 (7.7)	1640 (10.4)		415 (6.9)	999 (9.2)	
2	690 (8.7)	1779 (11.3)		513 (8.5)	1142 (10.5)	
3	753 (9.5)	1928 (12.2)		621 (10.3)	1204 (11.1)	
4	867 (11.0)	1696 (10.8)		732 (12.1)	1176 (10.8)	
5	871 (11.0)	1517 (9.6)		765 (12.6)	1191 (10.9)	
6	834 (10.6)	1305 (8.3)		745 (12.3)	1061 (9.7)	
7	666 (8.4)	956 (6.1)		596 (9.8)	788 (7.2)	
8	408 (5.2)	618 (3.9)		367 (6.1)	469 (4.3)	
9	160 (2.0)	263 (1.7)		145 (2.4)	204 (1.9)	
10	51 (0.6)	210 (1.3)		44 (0.7)	114 (1.0)	
Medication exposure						
Fentanyl and propofol coadministration, n (%)			<.001			—^d^
No	1661 (21.1)	4772 (30.3)		—	—	
Yes	6229 (78.9)	10,996 (69.7)		—	—	
Fentanyl dose (μg/kg), median (IQR)	1.47 (1.08-2.14)	1.60 (1.10-2.52)	<.001	1.47 (1.08-2.14)	1.60 (1.10-2.52)	<.001
Propofol dose (mm/kg), median (IQR)	2.13 (1.72-2.59)	2.00 (1.47-2.56)	<.001	2.13 (1.72-2.59)	2.00 (1.47-2.56)	<.001
Surgical characteristics						
Surgery type, n (%)			<.001			<.001
Cardiothoracic	252 (3.2)	1113 (7.1)		100 (1.7)	721 (6.6)	
Neurovascular	983 (12.5)	1867 (11.8)		281 (4.6)	1221 (11.2)	
Abdominopelvic	2185 (27.7)	3614 (22.9)		1984 (32.8)	2638 (24.2)	
Musculoskeletal	1765 (22.4)	2443 (15.5)		1606 (26.5)	2010 (18.5)	
Other	2705 (34.3)	6731 (42.7)		2087 (34.5)	4301 (39.5)	
Comorbidities, n (%)						
Hypertension			<.001			<.001
No	5890 (74.7)	11,378 (72.2)		4998 (82.5)	8044 (73.9)	
Yes	2000 (25.3)	4390 (27.8)		1060 (17.5)	2847 (26.1)	
Diabetes mellitus			.86			<.001
No	6866 (87.0)	13,709 (86.9)		5541 (91.5)	9635 (88.5)	
Yes	1024 (13.0)	2059 (13.1)		517 (8.5)	1256 (11.5)	
Hyperlipidemia			.48			<.001
No	6762 (85.7)	13,459 (85.4)		5452 (90.0)	9412 (86.4)	
Yes	1128 (14.3)	2309 (14.6)		606 (10.0)	1479 (13.6)	
Chronic kidney disease			<.001			<.001
No	6368 (80.7)	13-729 (87.1)		5447 (89.9)	9604 (88.2)	
Yes	1522 (19.3)	2039 (12.9)		611 (10.1)	1287 (11.8)	
Laboratory values, median (IQR)						
Hemoglobin	12.1 (11.30-13.70)	11.09 (9.14-12.80)	<.001	12.50 (11.53-13.90)	11.55 (9.46-13.08)	<.001
Neutrophil count	6.27 (4.80-7.85)	7.25 (5.16-10)	<.001	6.25 (4.75-8.16)	7.10 (5.10-9.76)	<.001
Serum chloride	103 (101-105)	104 (101-106.83)	<.001	103.50 (101.00-105.40)	103.72 (101-106)	<.001
Hematocrit	36.01 (33.98-40.42)	33.20 (27.28-38.10)	<.001	37.30 (34.50-41.19)	34.50 (28.15-38.90)	<.001
Platelet count	244 (206-292.50)	229 (174-296.50)	<.001	248 (207-299)	236.33 (184.13-303.44)	<.001
Serum potassium	3.93 (3.70-4.20)	3.93 (3.70-4.20)	.01	3.90 (3.70-4.14)	3.92 (3.70-4.19)	<.001
Blood glucose	109 (96-132)	130 (108.33-163)	<.001	109 (96.50-129.50)	125 (105-154.56)	<.001
Total carbon dioxide	25.07 (23.50-27)	24.50 (22.52-26.29)	<.001	25 (23.50-27)	24.75 (23-26.43)	<.001
Serum calcium	9.10 (8.77-9.40)	8.63 (8.10-9.10)	<.001	9.10 (8.75-9.40)	8.72 (8.20-9.17)	<.001
Serum creatinine	0.85 (0.70-1.29)	0.88 (0.68-1.30)	.65	0.80 (0.67-1)	0.84 (0.67-1.15)	<.001
Serum sodium	137 (135-138.33)	137 (134.93-139)	<.001	137 (135-138.33)	136.85 (134.80-138.65)	.04
Leukocyte count	8.84 (7.20-10.44)	9.71 (7.47-12.70)	<.001	8.84 (7.17-10.84)	9.60 (7.40-12.43)	<.001
Blood urea nitrogen	15 (11-23)	16.50 (11.89-26)	<.001	14 (10.50-19)	15.50 (11.24-23)	<.001
Erythrocyte count	4.24 (3.72-4.64)	3.79 (3.14-4.47)	<.001	4.31 (3.69-4.75)	3.88 (3.22-4.53)	<.001

a The doses of fentanyl and propofol in the total eligible cohort were not imputed.

b ICU: intensive care unit.

c ASA: American Society of Anesthesiologists

d Not applicable.

### Total Eligible Cohort Analysis

Primary dose-response analysis revealed clinically significant thresholds for ICU admission risk. The average treatment effect of 0.0102 indicated a 1.02% absolute increase in ICU admission risk associated with fentanyl-propofol combinations. The distribution of treatment effects showed significant right skewness, indicating that while most patients experience minimal increased risk, a subset faces substantially higher risk from drug combinations. The CDT identified serum creatinine as the primary splitting variable, with subsequent stratification by glucose, age, and calcium (along with erythrocyte count, ASA physical status, and electrolytes) defining patient subgroups with differential susceptibility.

### Dual-Exposure Cohort Analysis

**Figure 2.**
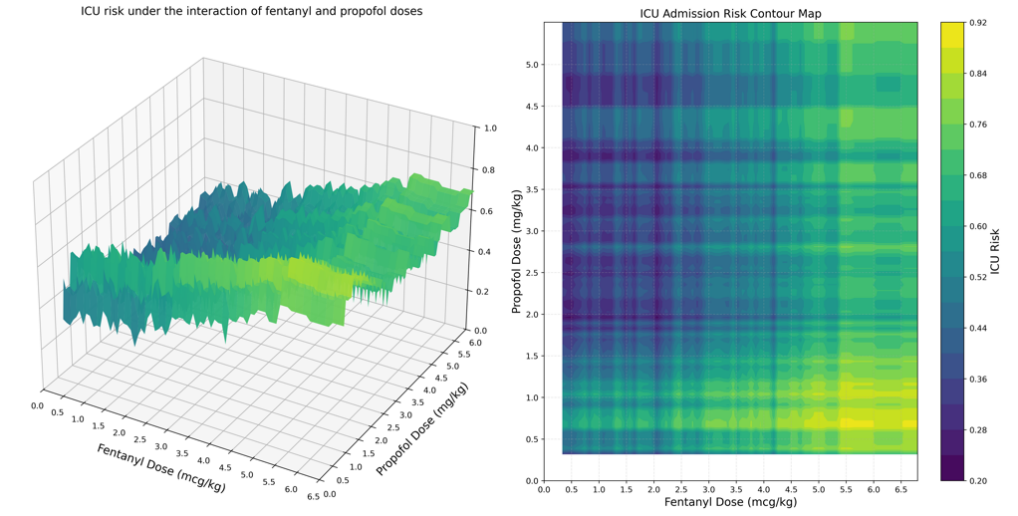
(Left) Intensive care unit (ICU) risk surface and (right) contour plot under fentanyl-propofol interaction.

Dose-response surface analysis identified distinct risk zones with immediate clinical applications (Figure 2). The average-dose region (fentanyl: 1.75‐2.0 mcg/kg and propofol: 2.1‐2.2 mg/kg) maintained ICU admission risk below 40%. The optimal dose range for decision-making was determined to be 3.5 to 4.0 mcg/kg for fentanyl and 1.25 to 4.25 mg/kg for propofol. In the moderate-dose region (fentanyl: 2.5‐3.5 mcg/kg and propofol: 2‐3 mg/kg), the ICU risk exhibited a nonlinear increase, gradually rising to 60%. Risk increased exponentially beyond these thresholds, reaching 92% for high-risk combinations (fentanyl >5 mcg/kg and propofol <1 mg/kg).

The 3D dose–response surface depicts the association between fentanyl dose (mcg/kg) on the x-axis, propofol dose (mg/kg) on the y-axis, and the model-predicted probability of ICU admission on the z-axis. The corresponding 2D contour plot presents the same dose ranges, with the color gradient indicating the predicted postoperative ICU admission risk.

Multivariate logistic regression analysis revealed a statistically significant positive interaction between fentanyl and propofol doses on the risk of ICU admission (interaction term *β*=.0243; *P*<.001). When the fentanyl dose exceeded 3 mcg/kg and the propofol dose exceeded 3.5 mg/kg, the synergistic effect between the 2 increased the predicted Δ risk of postoperative ICU admission by 36% (95% CI 0.351- 0.509; *P*<.001).

**Figure 3.**
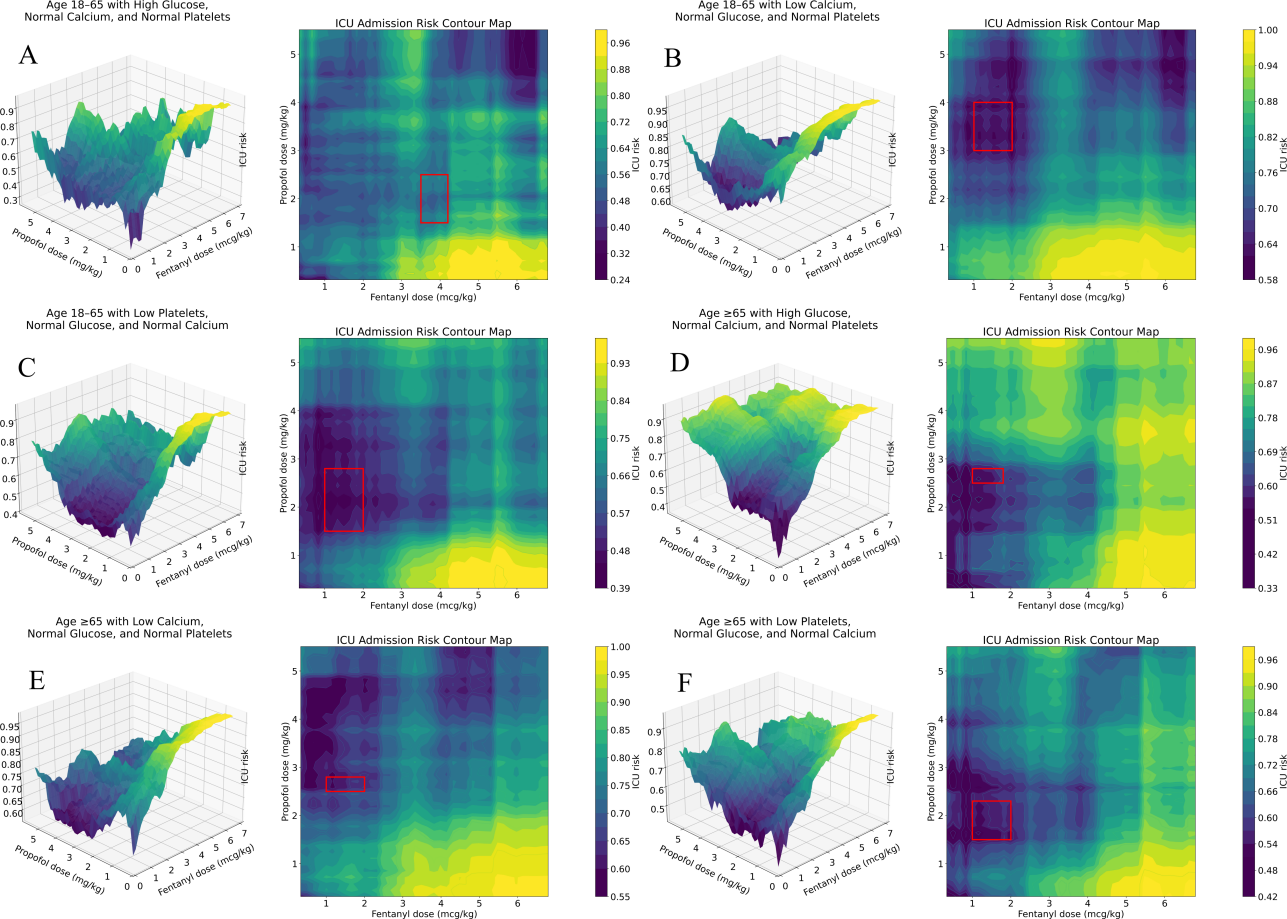
Dose-response surfaces and contour plots for sensitive groups.

Compared with the reference group, all 6 drug-sensitive groups (Figure 3) demonstrated a marked amplification and leftward shift of the risk gradient. In the 3D risk surface plot, this trend was marked by a steeper ascending slope in the low-dose region and an earlier onset of the high-risk plateau.

Figure 3A demonstrates that within the low-to-medium dose range, the early slope is steeper and the inflection point occurs at a lower combined dose. Figure 3B illustrates that the risk ridge along the fentanyl axis increases more abruptly, with the high-risk region compressed along the propofol axis and extended across a broader fentanyl range. In Figure 3C, the onset of risk occurs at lower doses and is accompanied by localized irregularities. Figure 3D shows that the risk surface flattens with increasing doses, with a gradual slope in the medium-dose range and an abrupt increase in the higher-dose region. Figure 3E exhibits a relatively flat surface, showing attenuated dose responsiveness and scattered high-risk plateaus. Figure 3F reveals a biphasic risk pattern, characterized by a rapid early increase followed by relative stabilization in the medium dose range.

### Interpretability

**Figure 4.**
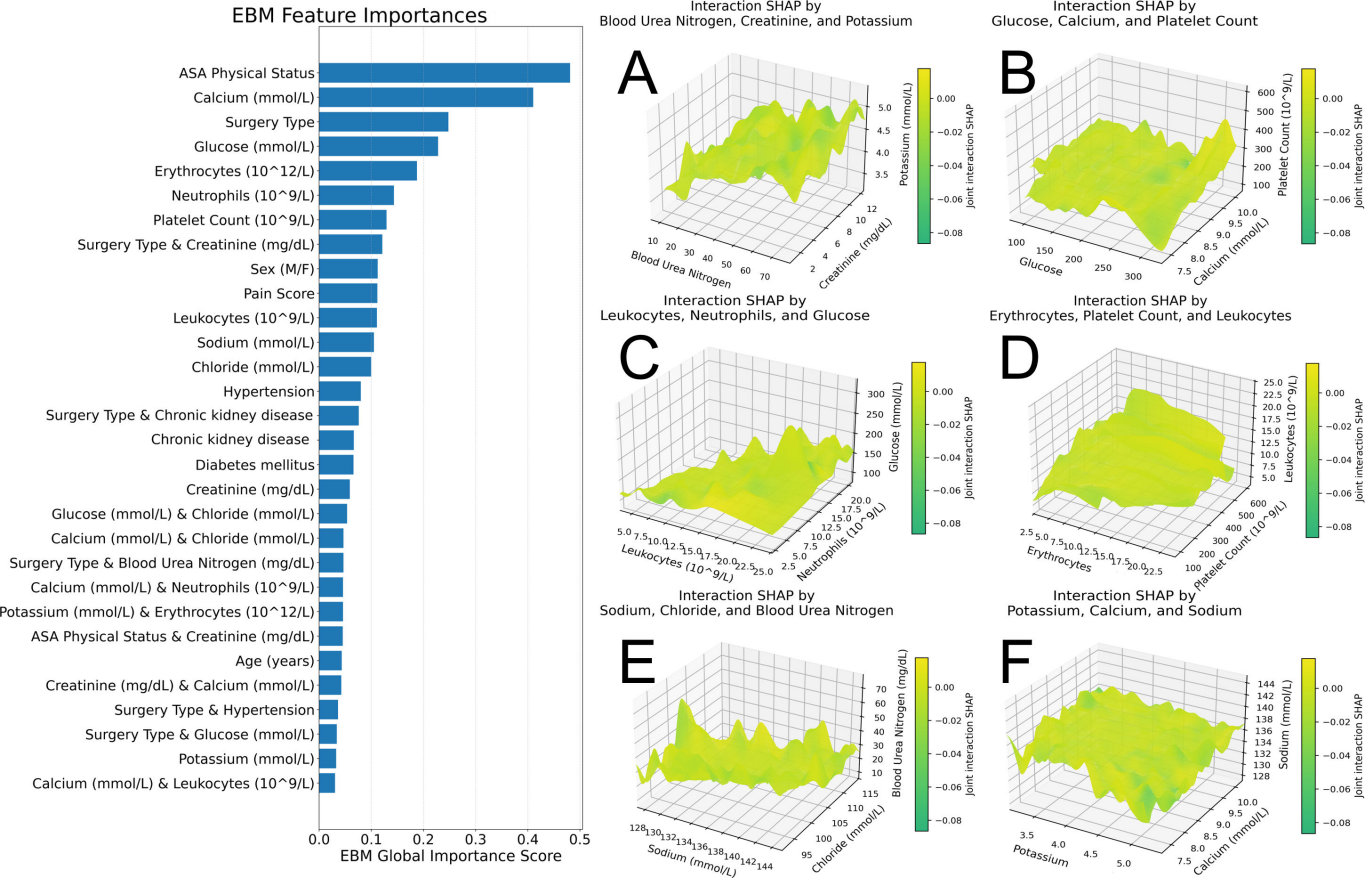
Global feature importance ranking from Explainable Boosting Machine (EBM) and group-specific Shapley additive explanation (SHAP) summary plots for intensive care unit (ICU) admission risk.

Figure 4 demonstrates that among all predictors, ASA physical status exhibits the highest overall ranking, with serum calcium and blood glucose also showing relatively high importance. The 3D SHAP interaction plot reveals that the interactive contribution of creatinine changes nonlinearly with blood urea nitrogen and potassium levels, with more pronounced effects observed at higher creatinine-to–blood urea nitrogen ratios. This pattern aligns with the early and marked increase in ICU risk depicted in the dose-response relationship.

The bar chart displays the average absolute importance scores of clinical features calculated using the Explainable Boosting Machine model. In each 3D graph, the z-axis represents the SHAP value of a specific clinical variable, quantifying its contribution to ICU admission risk prediction. The x-axis and y-axis correspond to 2 interacting features that jointly determine the magnitude and direction of the variable’s contribution.

## Discussion

### Principal Findings

This study provides a counterfactual modeling approach to analyzing fentanyl-propofol dose-response relationships and ICU admission risk, with potential implications for clinical decision support and hypothesis generation. Our findings demonstrate that current empirical dosing approaches may expose patients to preventable complications, while individualized risk assessment could substantially reduce ICU admissions and associated costs.

The clinical significance of these findings is rooted in the respiratory depressant effects of both agents. Propofol can impair ventilation via upper-airway collapsibility and central respiratory depression [6,20,21], while fentanyl further suppresses brain stem ventilatory control and, at higher opioid exposure or with rapid administration, may cause chest wall rigidity that impairs ventilation [22]. These complementary effects plausibly contribute to the nonlinear risk escalation and ridge-like high-risk zones observed in our dose-response surfaces, particularly at higher fentanyl doses combined with lower propofol doses.

In all subgroups, the higher the fentanyl exposure, the more sharply the ICU predicted risk rose, which may reflect the combined effect of cardiopulmonary depression and propofol-induced vasodilation and systemic vascular resistance reduction [23,24]. Pharmacodynamic synergy can alter anesthetic requirements, such that minor changes in opioid exposure can alter the propofol dose needed to achieve adequate sedation and cause patients to cross the hemodynamic threshold, which is consistent with steeper spines and earlier inflection points observed on the curve [25,26].

Older patients are more susceptible because of their reduced cardiovascular reserve and higher sensitivity to anesthesia-related hypotension [24]. The pattern in the hyperglycemic subgroup is consistent with evidence that stress hyperglycemia is associated with postoperative complications and mortality [27,28]. Similarly, hypocalcemia may reduce patients’ tolerance to intraoperative instability by affecting myocardial contractility, vascular tone, and coagulation function [29]. Finally, the elevated risk and irregular plateau in thrombocytopenia may reflect increased bleeding susceptibility and treatment escalation, as abnormal platelet counts and preoperative platelet transfusion are associated with higher ICU admission rates and longer hospital stays in noncardiac surgery [30,31].

In the dual-exposure cohort, as the doses of both drugs increase, the risk of ICU admission exhibits a nonlinear upward trend. When the fentanyl dose exceeds 3.5 mcg/kg, the predicted probability of ICU admission increases sharply, particularly when propofol doses are low to moderate rather than uniformly high, reaching 36% in the high-risk region and indicating a pronounced ridge-like dose-response gradient.

The asymmetric response pattern observed indicates that when propofol doses are below 3 mg/kg, incremental increases in propofol lead to a reduction in ICU admission risk. Conversely, across nearly all dose ranges, increasing fentanyl doses are associated with elevated ICU admission risks. This may be attributed to the relatively low frequency of the high-dose propofol+low-dose fentanyl combination in the sample, thereby constraining the model’s generalization capacity in this region and necessitating cautious interpretation.

Our findings offer specific guidance for clinical practice: ICU risk is minimized within a narrow corridor of combined fentanyl and propofol dosing, delineated by the red box, and centered on fentanyl doses of 3.5 to 4.0 mcg/kg and propofol doses of 1.25 to 4.25 mg/kg. These results challenge the assumption that higher propofol doses uniformly increase risk [6,15]. Beyond this optimal range, risk escalates nonlinearly, with the highest-risk zone occurring when fentanyl exceeds 5 mcg/kg while propofol remains low (<1 mg/kg) [32]. Therefore, the optimal dosing window for clinical decision-making was defined as fentanyl 3.5 to 4.0 mcg/kg and propofol 1.25 to 4.25 mg/kg.

### Clinical Practice Implications

These implications are exploratory and hypothesis-generating, as the findings arise from observational counterfactual modeling and require prospective validation before clinical implementation. The findings provide a foundation for transforming anesthetic dosing from empirical guidelines to evidence-based, individualized approaches. The identification of specific dose thresholds and patient populations considered vulnerable enables the development of real-time risk assessment tools incorporating patient characteristics that could be integrated into anesthesia information management systems [2,3]. This research establishes the foundation for transforming anesthetic dosing from empirical guidelines to precision medicine approaches; evidence-based dosing algorithms using patient-specific risk profiles could guide dosing decisions [3,10]. Enhanced monitoring protocols for high-risk patients could enable early intervention and help prevent progression to complications requiring ICU admission [2,3,10].

### Limitations

Several limitations must be acknowledged. The single-center design may restrict the generalizability of findings across diverse health care systems, patient case mixes, and perioperative protocols, highlighting the necessity for external validation. As an observational study, causal inferences are inherently susceptible to residual confounding. Furthermore, the maximum depth constraint of the CDT algorithm may overlook extended confounding pathways. Before clinical implementation, these results must be validated in independent cohorts and varied health care settings.

### Future Directions

This research establishes a foundation for several critical next steps: multicenter validation studies across diverse health care settings, interventional trials testing individualized dosing algorithms vs standard care, implementation research examining real-world application of risk assessment tools, technology integration with clinical decision support systems, and comprehensive cost-effectiveness studies.

### Conclusions

This study demonstrates that fentanyl-propofol combinations exhibit complex, nonlinear dose-response relationships with ICU admission risk. These findings require external validation before they can inform clinical decision support or guide future research. The optimal dose range for decision-making was determined to be 3.5 to 4.0 mcg/kg for fentanyl and 1.25 to 4.25 mg/kg for propofol. High-dose combinations (fentanyl >5 mcg/kg and propofol <1 mg/kg) increase the risk of ICU admission by 36%, whereas 6 distinct patient subgroups show differential vulnerability, necessitating individualized management approaches.

## Supplementary material

10.2196/80294 Multimedia Appendix 1 Supplementary tables and figures.

## Abbreviations

CDT: causal decision tree
ICU: intensive care unit
MOVER: Medical Informatics Operating Room Vitals and Events Repository
SHAP: Shapley additive explanation
