# Supplementary material for: Stratified Causal Inference for Intensive Care Unit Risk Prediction: Informatics-Based Modeling of Anesthetic Drug Combinations
Source: JMIR Form Res. 2026 Feb 25;10:e80294. doi: 10.2196/80294 (PMC12935416; doi:10.2196/80294)
Supplement: Multimedia Appendix 1 [file formative-v10-e80294-s001.docx]

Supplementary Table S1. Mapping of surgical procedure categories to representative procedure types.

| Surgery category | Specific procedure type |
| --- | --- |
| Cardiothoracic surgery | Coronary artery bypass grafting |
|  | Valve replacement |
|  | Valve repair |
|  | Thoracic aortic aneurysm repair |
|  | Aortic dissection repair |
|  | Thoracic surgery (lung resection) |
|  | Mediastinal surgery |
|  | Pericardial surgery |
| Neurovascular surgery | Craniotomy |
|  | Intracranial aneurysm clipping |
|  | Intracranial aneurysm endovascular coiling |
|  | Carotid endarterectomy |
|  | Carotid artery stenting |
|  | Cerebral revascularization |
|  | Spine decompression (neurosurgical) |
| Abdominopelvic surgery | Exploratory laparotomy |
|  | Bowel resection |
|  | Appendectomy |
|  | Cholecystectomy |
|  | Gastrectomy |
|  | Hepatectomy |
|  | Pancreatectomy |
|  | Nephrectomy |
|  | Cystectomy |
|  | Hysterectomy |
|  | Caesarean delivery |
|  | Prostatectomy |
| Musculoskeletal surgery | Total hip arthroplasty |
|  | Total knee arthroplasty |
|  | Hip fracture fixation |
|  | Long-bone fracture fixation |
|  | Spinal fusion (orthopaedic) |
|  | Laminectomy (orthopaedic) |
|  | Shoulder arthroplasty |
|  | Arthroscopic ligament reconstruction |
| Other | Otolaryngology surgery |
|  | Ophthalmic surgery |
|  | Plastic and reconstructive surgery |
|  | Minor general surgery |
|  | Interventional radiology procedures |
|  | Endoscopy |

Supplementary Table S2. ICD-9-CM and ICD-10-CM code definitions for chronic comorbidities.

| Chronic condition | Codes |
| --- | --- |
| Hypertension | ICD-10-CM: I10; I11.*; I12.*; I13.*; I15.* \| ICD-9-CM: 401.*; 402.*; 403.*; 404.*; 405.* |
| Diabetes mellitus | ICD-10-CM: E08.*; E09.*; E10.*; E11.*; E13.* \| ICD-9-CM: 250.* |
| Hyperlipidaemia | ICD-10-CM: E78.* (E78.0–E78.5, E78.89, E78.9) \| ICD-9-CM: 272.* |
| Chronic kidney disease | ICD-10-CM: N18.*; N19; I12.*; I13.*; Z99.2 \| ICD-9-CM: 585.*; 586; 403.*; 404.*; V45.1; V56.* |

Supplementary Table S3. Data completeness.

| Variable | Total eligible cohort missing n (%) | Dual-exposure cohort missing n (%) | Missing >20% in either cohort |
| --- | --- | --- | --- |
| Demographics | | | |
| Age | 0 (0.00%) | 0 (0.00%) | No |
| Sex | 0 (0.00%) | 0 (0.00%) | No |
| Body weight (kg) | 377 (1.59%) | 0 (0.00%) | No |
| Physiological parameters | | | |
| ASA physical status | 0 (0%) | 0 (0 %) | No |
| Pain score | 0 (0%) | 0 (0%) | No |
| Surgical characteristics | | | |
| Surgery type category | 0 (0.00%) | 0 (0.00%) | No |
| Comorbidities | | | |
| Hypertension | 0 (0.00%) | 0 (0.00%) | No |
| Diabetes mellitus | 0 (0.00%) | 0 (0.00%) | No |
| Hyperlipidaemia | 0 (0.00%) | 0 (0.00%) | No |
| Chronic kidney disease | 0 (0.00%) | 0 (0.00%) | No |
| Medication exposure | | | |
| Fentanyl dose (micrograms per kilogram) | 6709 (28.36%) | 0 (0.00%) | - |
| Propofol dose (milligrams per kilogram) | 6709 (28.36%) | 0 (0.00%) | - |
| Laboratory values | | | |
| Haemoglobin | 1886 (7.97%) | 1032 (6.09%) | No |
| Haematocrit | 1904 (8.05%) | 1046 (6.17%) | No |
| Erythrocyte count | 1920 (8.12%) | 1057 (6.24%) | No |
| Leukocyte count | 1930 (8.16%) | 1066 (6.29%) | No |
| Neutrophil count | 4410 (18.64%) | 2860 (16.87%) | No |
| Platelet count | 1966 (8.31%) | 1093 (6.45%) | No |
| Serum sodium | 93 (0.39%) | 54 (0.32%) | No |
| Serum potassium | 44 (0.19%) | 40 (0.24%) | No |
| Serum chloride | 264 (1.12%) | 155 (0.91%) | No |
| Blood urea nitrogen | 269 (1.14%) | 157 (0.93%) | No |
| Serum creatinine | 272 (1.15%) | 158 (0.93%) | No |
| Blood glucose | 95 (0.40%) | 55 (0.32%) | No |
| Total carbon dioxide | 1750 (7.40%) | 925 (5.46%) | No |
| Serum calcium | 1746 (7.38%) | 922 (5.44%) | No |
| Serum albumin | 6710 (28.36%) | 4381 (25.85%) | Yes |
| Total bilirubin | 7501 (31.71%) | 4223 (24.92%) | Yes |
| Alanine aminotransferase | 6783 (28.67%) | 3706 (21.87%) | Yes |
| Aspartate aminotransferase | 7141 (30.18%) | 5027 (29.66%) | Yes |
| Serum lactate | 7274 (30.75%) | 4977 (29.36%) | Yes |
| International normalised ratio | 7483 (31.63%) | 5172 (30.52%) | Yes |

Supplementary Table S4. Collinearity results.

| Variable | VIF |
| --- | --- |
| Age | 1.48 |
| Sex = Male | 1.18 |
| Body weight (kg) | 1.26 |
| ASA physical status = 2 | 4.47 |
| ASA physical status = 3 | 3.96 |
| ASA physical status = 4 | 3.76 |
| ASA physical status = 5 | 1.16 |
| Pain score = 1 | 1.30 |
| Pain score = 2 | 1.36 |
| Pain score = 3 | 1.44 |
| Pain score = 4 | 1.50 |
| Pain score = 5 | 1.56 |
| Pain score = 6 | 1.55 |
| Pain score = 7 | 1.47 |
| Pain score = 8 | 1.29 |
| Pain score = 9 | 1.14 |
| Pain score = 10 | 1.07 |
| Surgery type = Cardiothoracic surgery | 1.27 |
| Surgery type = Neurovascular surgery | 1.35 |
| Surgery type = Musculoskeletal surgery | 1.55 |
| Surgery type = Other | 1.59 |
| Hypertension = Yes | 1.74 |
| Diabetes mellitus = Yes | 1.41 |
| Hyperlipidaemia = Yes | 1.40 |
| Chronic kidney disease = Yes | 2.03 |
| Haemoglobin | 33.52 |
| Haematocrit | 32.93 |
| Erythrocyte count | 1.03 |
| Leukocyte count | 3.02 |
| Neutrophil count | 3.28 |
| Platelet count | 1.24 |
| Serum sodium | 4.17 |
| Serum potassium | 1.46 |
| Serum chloride | 4.37 |
| Serum calcium | 1.40 |
| Blood glucose | 1.29 |
| Total carbon dioxide | 2.44 |
| Blood urea nitrogen | 2.26 |
| Serum creatinine | 1.12 |

Haemoglobin and Haematocrit were excluded.

Supplementary Table S5. Variable dictionary.

| **Variable** | **Category** | **Clinical Explanation** |
| --- | --- | --- |
| Age | Confounder | Key demographic factor influencing pharmacokinetics and physiological reserve. |
| Blood Urea Nitrogen (mg/dL) | Confounder | Renal function marker, affected by hydration and protein metabolism. |
| Calcium (mmol/L) | Confounder | Serum calcium level, reflects metabolic and neuromuscular stability. |
| Chloride (mmol/L) | Confounder | Electrolyte balance marker, relates to acid-base homeostasis. |
| Creatinine (mg/dL) | Confounder | Kidney function indicator, elevated levels suggest renal impairment. |
| Erythrocytes (10^12/L) | Confounder | Red blood cell count, related to oxygen-carrying capacity. |
| Glucose (mmol/L) | Confounder | Blood glucose level, can indicate metabolic stress or diabetes. |
| Leukocytes (10^9/L) | Confounder | Total white blood cell count, marker of immune response. |
| Neutrophils (10^9/L) | Confounder | Major white blood cell type, indicates inflammatory or infectious states. |
| Pain Score | Confounder | Subjective measure of patient discomfort, may influence sedative needs. |
| Platelet Count (10^9/L) | Confounder | Essential for clotting function, low counts increase bleeding risk. |
| Sex (M/F) | Confounder | Biological sex, associated with differential baseline risks and drug metabolism. |
| Surgery Type | Confounder | Procedure category reflecting surgical complexity and perioperative physiological stress. |
| Potassium (mmol/L) | Confounder | Crucial for cardiac and neuromuscular function. |
| Sodium (mmol/L) | Confounder | Key extracellular electrolyte, affects fluid balance and nerve function. |
| ASA Physical Status | Confounder | Preoperative physical status classification summarizing overall comorbidity burden and baseline physiological reserve; associated with perioperative risk and anaesthetic dosing decisions. |
| Hypertension | Confounder | History of hypertension reflects chronic cardiovascular risk and vascular stiffness; may influence intraoperative haemodynamic and postoperative complications. |
| Diabetes mellitus | Confounder | Diabetes indicates chronic metabolic dysregulation and micro/macrovascular disease; affects perioperative stress response, infection risk, and recovery. |
| Hyperlipidaemia | Confounder | Hyperlipidaemia is a marker of atherosclerotic risk and metabolic syndrome; associated with baseline cardiovascular risk and perioperative outcomes. |
| Chronic kidney disease (Yes/No) | Confounder | CKD reflects impaired renal clearance and systemic comorbidity; influences drug pharmacokinetics, fluid/electrolyte balance, and postoperative risk. |
| Fentanyl Dose | Treatment | Intraoperative opioid dose for analgesia. |
| Propofol Dose | Treatment | Intraoperative sedative/hypnotic dose. |
| ICU Admission | Outcome | Primary outcome, reflects severity and postoperative recovery trajectory. |

Supplementary Table S6. Mode table of normal range reference values in the total eligible cohort.

| Index | Range | Female | Male |
| --- | --- | --- | --- |
| Potassium | 25.0-125.0 | 0 | 3 |
| Potassium.1 | 3.5-5.1 | 8231 | 12627 |
| Potassium.2 | 3.7-5.5 | 1080 | 1673 |
| Sodium | 136-145 | 7876 | 11810 |
| Sodium.1 | 138-146 | 1416 | 2463 |
| Glucose | 41-70 | 53 | 88 |
| Glucose.1 | 70-110 | 1455 | 2536 |
| Glucose.2 | 70-115 | 3665 | 5506 |
| Glucose.3 | 85-125 | 4117 | 6143 |
| Chloride | 98-107 | 8353 | 12920 |
| Chloride.1 | 99-111 | 864 | 1257 |
| Urea nitrogen | 7.0-25.0 | 9217 | 14172 |
| Creatinine | 0.0-7.0 | 0 | 1 |
| Creatinine.1 | 0.6-1.2 | 8520 | 0 |
| Creatinine.2 | 0.6-1.3 | 692 | 912 |
| Creatinine.3 | 0.7-1.3 | 2 | 13253 |
| Creatinine.4 | 1.5-2.0 | 3 | 3 |
| Calcium | 8.6-10.3 | 8569 | 13343 |
| Carbon dioxide | 16-21 | 14 | 19 |
| Carbon dioxide.1 | 21-31 | 8551 | 13324 |
| Hemoglobin | 11.5-15.0 | 8473 | 0 |
| Hemoglobin.1 | 12.0-17.0 | 32 | 87 |
| Hemoglobin.2 | 13.0-16.0 | 0 | 19 |
| Hemoglobin.3 | 13.5-16.9 | 2 | 13159 |
| Hematocrit | 34.0-44.0 | 8331 | 0 |
| Hematocrit.1 | 37.0-49.0 | 0 | 19 |
| Hematocrit.2 | 38-46 | 144 | 0 |
| Hematocrit.3 | 38-51 | 23 | 56 |
| Hematocrit.4 | 39.5-50.0 | 2 | 12915 |
| Hematocrit.5 | 43-51 | 0 | 264 |
| Erythrocytes | 0-3 | 1108 | 1487 |
| Erythrocytes.1 | 3.70-5.00 | 7381 | 801 |
| Erythrocytes.2 | 4.38-5.62 | 0 | 10961 |
| Leukocytes | 4.0-10.5 | 8471 | 13224 |
| Leukocytes.1 | 4.5-13.5 | 14 | 19 |
| Platelets | 150-400 | 8457 | 13202 |
| Platelets.1 | 150-450 | 14 | 19 |
| Neutrophils | 1.8-8.0 | 8 | 14 |
| Neutrophils.1 | 2.0-7.5 | 72 | 101 |
| Neutrophils.2 | 2.0-8.1 | 7583 | 11470 |

Supplementary Table S7. Mode table of normal range reference values in the dual-exposure cohort.

| Index | Reference | Female | Male |
| --- | --- | --- | --- |
| Hemoglobin | 11.5-15.0 | 6454 | 0 |
| Hemoglobin.1 | 12.0-17.0 | 11 | 20 |
| Hemoglobin.2 | 13.0-16.0 | 0 | 16 |
| Hemoglobin.3 | 13.5-16.9 | 1 | 9633 |
| Neutrophils | 1.8-8.0 | 8 | 11 |
| Neutrophils.1 | 2.0-7.5 | 40 | 55 |
| Neutrophils.2 | 2.0-8.1 | 5789 | 8328 |
| Chloride | 98-107 | 6416 | 9523 |
| Chloride.1 | 99-111 | 459 | 662 |
| Hematocrit | 34.0-44.0 | 6383 | 0 |
| Hematocrit.1 | 37.0-49.0 | 0 | 16 |
| Hematocrit.2 | 38-46 | 71 | 0 |
| Hematocrit.3 | 38-51 | 7 | 14 |
| Hematocrit.4 | 39.5-50.0 | 1 | 9514 |
| Hematocrit.5 | 43-51 | 0 | 115 |
| Platelets | 150-400 | 6423 | 9620 |
| Platelets.1 | 150-450 | 14 | 16 |
| Potassium | 3.5-5.1 | 6351 | 9388 |
| Potassium.1 | 3.7-5.5 | 578 | 867 |
| Glucose | 41-70 | 28 | 47 |
| Glucose.1 | 70-110 | 765 | 1323 |
| Glucose.2 * | 70-115 | 3032 | 4458 |
| Glucose.3 * | 85-125 | 3097 | 4415 |
| Carbon dioxide | 16-21 | 14 | 16 |
| Carbon dioxide.1 | 21-31 | 6501 | 9715 |
| Calcium_ | 8.6-10.3 | 6518 | 9732 |
| Creatinine | 0.6-1.2 | 6488 | 0 |
| Creatinine.1 | 0.6-1.3 | 385 | 505 |
| Creatinine.2 | 0.7-1.3 | 1 | 9675 |
| Creatinine.3 | 1.5-2.0 | 1 | 2 |
| Sodium | 136-145 | 6180 | 8948 |
| Sodium.1 | 138-146 | 743 | 1295 |
| Leukocytes | 4.0-10.5 | 6435 | 9635 |
| Leukocytes.1 | 4.5-13.5 | 14 | 16 |
| Urea nitrogen | 7.0-25.0 | 6875 | 10183 |
| Erythrocytes | 0-3 | 835 | 1048 |
| Erythrocytes.1 | 3.70-5.00 | 5617 | 527 |
| Erythrocytes.2 | 4.38-5.62 | 0 | 8082 |

* The frequency differences in glucose among the quantities are very small. To maintain consistency among the cohorts, we choose to use the mode of the total eligible cohort for the sake of comparability, which is 85-125 as the normal range.

Supplementary Table S8. Distribution of primary surgical procedures in the total eligible cohort. Only display the names of surgeries with a sample size of 50 or more.

| **Procedure** | **Count** | **Proportion(%)** |
| --- | --- | --- |
| Laparotomy, exploratory | 693 | 0.029 |
| Cholecystectomy, laparoscopic | 662 | 0.027 |
| Debridement, with split-thickness skin graft application | 632 | 0.026 |
| Laparoscopy, diagnostic | 569 | 0.024 |
| Irrigation and debridement, lower extremity | 522 | 0.022 |
| Appendectomy, laparoscopic | 491 | 0.02 |
| Av fistulogram, with angioplasty if indicated | 465 | 0.019 |
| Orif, Fracture, femur | 435 | 0.018 |
| Creation, Av fistula | 379 | 0.016 |
| Transplant recipient, kidney, from deceased donor | 375 | 0.016 |
| Debridement, wound | 371 | 0.015 |
| Irrigation and debridement, extremity | 365 | 0.015 |
| Cabg (coronary artery bypass graft) | 345 | 0.014 |
| Orif, ankle | 313 | 0.013 |
| Orif, fracture, tibia | 306 | 0.013 |
| Egd (esophagogastroduodenoscopy) | 224 | 0.009 |
| Laparoscopy, diagnostic, with exploratory laparotomy if indicated | 222 | 0.009 |
| Catheterization, heart, left, with intervention if indicated | 215 | 0.009 |
| Tracheostomy | 201 | 0.008 |
| Angiogram, lower extremity, with Angioplasty | 198 | 0.008 |
| Amputation, below knee | 197 | 0.008 |
| Allograft, skin | 184 | 0.008 |
| Irrigation and debridement, wound | 184 | 0.008 |
| Craniotomy | 180 | 0.007 |
| Irrigation and debridement, upper extremity | 170 | 0.007 |
| Cholecystectomy, laparoscopic, with cholangiogram | 164 | 0.007 |
| Orif, fracture, radius | 163 | 0.007 |
| Irrigation and debridement, hand | 161 | 0.007 |
| Orif, fracture, humerus | 158 | 0.007 |
| Egd, with biopsy | 156 | 0.006 |
| Craniectomy or craniotomy, emergent | 154 | 0.006 |
| Amputation, toe | 152 | 0.006 |
| Insertion, drain | 144 | 0.006 |
| Gi egd eus fna | 138 | 0.006 |
| Bronchoscopy, rigid | 137 | 0.006 |
| Cystoscopy, with retrograde pyelogram and ureteral stent insertion | 137 | 0.006 |
| Exam under anesthesia, anorectal | 135 | 0.006 |
| Orif, fracture, acetabulum | 130 | 0.005 |
| Irrigation, wound, abdomen | 129 | 0.005 |
| Angiogram, vessel, cerebral, with embolization | 123 | 0.005 |
| Craniotomy or burr hole, with subdural hematoma evacuation | 123 | 0.005 |
| Ercp (endoscopic retrograde cholangiopancreatography) | 123 | 0.005 |
| Exploration, neck | 118 | 0.005 |
| Repair, globe, ruptured | 115 | 0.005 |
| Ir angio cerebral diagnostic | 115 | 0.005 |
| Laparotomy, exploratory, emergent for abdominal trauma | 114 | 0.005 |
| Ir plcmt gastrostomy tube | 108 | 0.004 |
| Creation, Av fistula, using vein transposition technique | 108 | 0.004 |
| Ir plcmt tunneled cath | 107 | 0.004 |
| Fusion, spine, lumbar and lumbosacral, using posterior technique | 105 | 0.004 |
| Insertion, catheter, for peritoneal dialysis | 105 | 0.004 |
| Gastrostomy, percutaneous, endoscopic | 103 | 0.004 |
| Angiogram, cerebral | 99 | 0.004 |
| Vats, with thoracotomy if indicated | 97 | 0.004 |
| Craniotomy, for brain neoplasm excision, using navigation system | 95 | 0.004 |
| Craniotomy, with neoplasm excision | 93 | 0.004 |
| Fixation, rib, using plate | 91 | 0.004 |
| Closed reduction, fracture, pelvis | 91 | 0.004 |
| Laparotomy, exploratory, with bowel resection | 89 | 0.004 |
| Exploration, upper extremity | 89 | 0.004 |
| Hemiarthroplasty, hip | 88 | 0.004 |
| Cystoscopy, with retrograde pyelogram, ureteroscopy, urinary Calculus laser lithotripsy, stent insert | 86 | 0.004 |
| Gi endoscopic ultrasound upper | 85 | 0.004 |
| Fusion, spine, cervical, posterior approach | 81 | 0.003 |
| Angiogram, with angioplasty | 80 | 0.003 |
| Placement, shunt, ventriculoperitoneal | 80 | 0.003 |
| Ir embo non-cns | 78 | 0.003 |
| Orif, fracture, tibia or fibula | 78 | 0.003 |
| Craniotomy, for hematoma evacuation | 77 | 0.003 |
| Exploration, chest | 75 | 0.003 |
| Ir nephrostomy tube | 75 | 0.003 |
| Video-assisted thoracoscopic surgery (vats) | 73 | 0.003 |
| Irrigation and debridement, knee | 73 | 0.003 |
| Dilation and evacuation, uterus | 71 | 0.003 |
| Extraction, cataract, with iol and glaucoma implant insertion | 71 | 0.003 |
| Angiogram | 71 | 0.003 |
| Exploration, wound | 70 | 0.003 |
| Exam under anesthesia, gynecologic | 68 | 0.003 |
| Insertion, tunneled central venous device, with port | 68 | 0.003 |
| Transplant recipient, kidney, from living donor | 67 | 0.003 |
| Repair, retinal detachment | 66 | 0.003 |
| Dilation and curettage | 66 | 0.003 |
| Orif, fracture, tibia, plateau | 66 | 0.003 |
| Replacement, aortic valve | 65 | 0.003 |
| Fusion, spine, thoracic or lumbar, or both thoracic and Lumbar, posterior approach, using instrumentation | 65 | 0.003 |
| Ir plcmt ivc filter | 64 | 0.003 |
| Fusion, spine, thoracic | 64 | 0.003 |
| Craniotomy, for subdural hematoma evacuation | 63 | 0.003 |
| Egd, with gastrojejunostomy tube insertion, percutaneous | 62 | 0.003 |
| Exam under anesthesia | 61 | 0.003 |
| Orif, fracture, radius or ulna | 59 | 0.002 |
| Revision, amputation site | 57 | 0.002 |
| Ir angio abdominal | 56 | 0.002 |
| Irrigation and debridement, foot | 56 | 0.002 |
| Insertion, tunneled cuffed hemodialysis catheter or tunneled Central venous catheter with port | 56 | 0.002 |
| Incision and drainage, abscess, upper extremity | 55 | 0.002 |
| External fixation, ankle | 55 | 0.002 |
| Orif posterior pelvis | 54 | 0.002 |
| Orif, fracture, mandible, with maxillomandibular fixation | 54 | 0.002 |
| Bronchoscopy, with ebus and biopsy | 53 | 0.002 |
| Fistulogram | 53 | 0.002 |
| Creation, colostomy or ileostomy, laparoscopic | 52 | 0.002 |
| Craniotomy, for suboccipital neoplasm | 51 | 0.002 |
| Orif, fracture, radius, distal | 51 | 0.002 |
| Craniectomy | 50 | 0.002 |

Supplementary Table S9. Distribution of primary surgical procedures in the dual-exposure cohort. Only display the names of surgeries with a sample size of 50 or more.

| **Procedure** | **Count** | **Proportion(%)** |
| --- | --- | --- |
| Cholecystectomy, laparoscopic | 622 | 0.036 |
| Laparoscopy, diagnostic | 522 | 0.03 |
| Debridement, with split-thickness skin graft application | 503 | 0.029 |
| Appendectomy, laparoscopic | 467 | 0.027 |
| Irrigation and debridement, Lower extremity | 429 | 0.025 |
| Transplant recipient, kidney, from deceased donor | 371 | 0.021 |
| Orif, fracture, femur | 371 | 0.021 |
| Laparotomy, exploratory | 367 | 0.021 |
| Irrigation and debridement, extremity | 302 | 0.017 |
| Orif, fracture, tibia | 275 | 0.016 |
| Orif, ankle | 272 | 0.016 |
| Debridement, wound | 269 | 0.016 |
| Cabg (coronary artery bypass graft) | 245 | 0.014 |
| Laparoscopy, diagnostic, with exploratory laparotomy if indicated | 191 | 0.011 |
| Cholecystectomy, laparoscopic, with cholangiogram | 155 | 0.009 |
| Irrigation and debridement, wound | 150 | 0.009 |
| Craniotomy | 144 | 0.008 |
| Egd (esophagogastroduodenoscopy) | 144 | 0.008 |
| Orif, fracture, radius | 142 | 0.008 |
| Irrigation and debridement, upper extremity | 141 | 0.008 |
| Orif, fracture, humerus | 138 | 0.008 |
| Amputation, below knee | 136 | 0.008 |
| Allograft, skin | 129 | 0.007 |
| Irrigation and debridement, hand | 128 | 0.007 |
| Cystoscopy, with retrograde pyelogram and ureteral stent insertion | 126 | 0.007 |
| Exam under anesthesia, anorectal | 117 | 0.007 |
| Gi egd eus fna | 117 | 0.007 |
| Orif, Fracture, acetabulum | 115 | 0.007 |
| Av fistulogram, with angioplasty if indicated | 115 | 0.007 |
| Repair, globe, ruptured | 106 | 0.006 |
| Angiogram, lower extremity, with angioplasty | 106 | 0.006 |
| Ercp (endoscopic retrograde cholangiopancreatography) | 102 | 0.006 |
| Craniotomy or burr hole, with subdural hematoma evacuation | 100 | 0.006 |
| Creation, av fistula | 97 | 0.006 |
| Egd, with biopsy | 95 | 0.005 |
| Irrigation, wound, abdomen | 94 | 0.005 |
| Fusion, spine, lumbar and lumbosacral, using posterior technique | 92 | 0.005 |
| Insertion, catheter, for peritoneal dialysis | 88 | 0.005 |
| Craniotomy, for brain neoplasm excision, using navigation system | 86 | 0.005 |
| Vats, with thoracotomy if indicated | 82 | 0.005 |
| Exploration, neck | 81 | 0.005 |
| Exploration, upper extremity | 80 | 0.005 |
| Craniotomy, with neoplasm excision | 77 | 0.004 |
| Cystoscopy, with retrograde pyelogram, ureteroscopy, urinary calculus laser lithotripsy, stent insert | 75 | 0.004 |
| Closed reduction, fracture, pelvis | 75 | 0.004 |
| Hemiarthroplasty, hip | 75 | 0.004 |
| Fixation, rib, using plate | 74 | 0.004 |
| Orif, fracture, tibia or fibula | 73 | 0.004 |
| Gi endoscopic ultrasound upper | 71 | 0.004 |
| Gastrostomy, percutaneous, endoscopic | 70 | 0.004 |
| Fusion, spine, cervical, posterior approach | 70 | 0.004 |
| Bronchoscopy, rigid | 69 | 0.004 |
| Amputation, toe | 69 | 0.004 |
| Tracheostomy | 67 | 0.004 |
| Placement, shunt, ventriculoperitoneal | 66 | 0.004 |
| Transplant recipient, kidney, from living donor | 66 | 0.004 |
| Craniectomy or craniotomy, emergent | 64 | 0.004 |
| Irrigation and debridement, knee | 63 | 0.004 |
| Extraction, cataract, with iol and glaucoma implant insertion | 61 | 0.004 |
| Repair, retinal detachment | 60 | 0.003 |
| Laparotomy, exploratory, emergent for abdominal trauma | 60 | 0.003 |
| Dilation and evacuation, uterus | 59 | 0.003 |
| Video-assisted thoracoscopic surgery (vats) | 57 | 0.003 |
| Orif, fracture, tibia, plateau | 57 | 0.003 |
| Fusion, spine, thoracic or Lumbar, or both thoracic and Lumbar, posterior approach, using instrumentation | 54 | 0.003 |
| Fusion, spine, thoracic | 53 | 0.003 |
| Orif, fracture, radius or Ulna | 52 | 0.003 |
| Exam under anesthesia, gynecologic | 52 | 0.003 |
| External fixation, ankle | 50 | 0.003 |
| Exploration, wound | 50 | 0.003 |

Supplementary Table S10. Model configuration

| **Purpose** | **Dataset** | **Model Name** | **Parameters** |
| --- | --- | --- | --- |
| Propensity score modeling for intraoperative fentanyl use | The dual-exposure cohort | XGBoost Regressor (Fentanyl PS) | {'subsample': 0.8, 'n_estimators': 200, 'max_depth': 3, 'learning_rate': 0.05, 'colsample_bytree': 1.0} |
| Propensity score modeling for intraoperative propofol use | The dual-exposure cohort | XGBoost Regressor (Propofol PS) | {'subsample': 0.8, 'n_estimators': 100, 'max_depth': 3, 'learning_rate': 0.05, 'colsample_bytree': 0.8} |
| Predicting ICU admission for downstream causal inference | The dual-exposure cohort | XGBoost Regressor (ATE model) | {'subsample': 0.8, 'n_estimators': 200, 'max_depth': 4, 'learning_rate': 0.05, 'colsample_bytree': 0.8} |
| Propensity score modeling for binary fentanyl-propofol combination | The total eligible cohort | XGBoost Classifier (Combo PS) | {'subsample': 1.0, 'n_estimators': 200, 'max_depth': 3, 'learning_rate': 0.05, 'colsample_bytree': 0.8} |
| Estimating ICU effect of combo therapy using Causal Forest | The total eligible cohort | XGBoost Regressor (Combo ATE) | {'subsample': 0.8, 'n_estimators': 100, 'max_depth': 4, 'learning_rate': 0.05, 'colsample_bytree': 1.0} |
| Joint dose prediction for fentanyl and propofol using multi-output regression | The dual-exposure cohort | MultiOutput XGBoost Regressor (Joint PS Residual) | {'estimator__n_estimators': 300, 'estimator__max_depth': 4, 'estimator__learning_rate': 0.01} |
| Joint dose prediction for fentanyl and propofol using multi-output regression | The dual-exposure cohort | MultiOutput Random Forest Regressor (Joint PS Residual) | {'estimator__max_depth': 6, 'estimator__n_estimators': 200} |
| Joint dose prediction for fentanyl and propofol using multi-output regression | The dual-exposure cohort | MultiOutput Ridge Regressor (Joint PS Residual) | {'estimator__alpha': 10.0} |
| Joint dose prediction for fentanyl and propofol using multi-output regression | The dual-exposure cohort | MultiOutput LightGBM Regressor (Joint PS Residual) | {'estimator__learning_rate': 0.05, 'estimator__n_estimators': 100, 'estimator__num_leaves': 15} |
| Joint dose prediction for fentanyl and propofol using multi-output regression | The dual-exposure cohort | MultiOutput CatBoost Regressor (Joint PS Residual) | {'estimator__depth': 6, 'estimator__iterations': 200, 'estimator__learning_rate': 0.05} |
| Joint dose prediction for fentanyl and propofol using multi-output regression | The dual-exposure cohort | MultiOutput KNN Regressor (Joint PS Residual) | {'estimator__n_neighbors': 9, 'estimator__weights': 'uniform'} |
| Joint dose prediction for fentanyl and propofol using multi-output regression | The dual-exposure cohort | MultiOutput SVR (Joint PS Residual) | {'estimator__C': 10.0, 'estimator__epsilon': 0.2, 'estimator__gamma': 'scale', 'estimator__kernel': 'rbf'} |
| Joint dose prediction for fentanyl and propofol using multi-output regression | The dual-exposure cohort | MultiOutput MLP Regressor (Joint PS Residual) | {'estimator__alpha': 0.0001, 'estimator__hidden_layer_sizes': (64, 32), 'estimator__learning_rate_init': 0.01} |
| Joint dose prediction for fentanyl and propofol using multi-output regression | The dual-exposure cohort | MultiOutput HistGradientBoosting Regressor (Joint PS Residual) | {'estimator__min_samples_leaf': 20, 'estimator__max_iter': 150, 'estimator__max_depth': 5, 'estimator__learning_rate': 0.05, 'estimator__l2_regularization': 0.1} |
| Residual-based GPS weight extraction using pre-trained SVR joint dose model | The dual-exposure cohort | SVR Regressor (Pretrained, Residual-GPS Weighting) | Loaded from 'best_joint_dose_model_svr.pkl' with previously tuned SVR parameters |
| Estimating patient-specific ICU admission effects of combo treatment using Causal Forest + CDT | Train data & Val data | CausalForestDML + DecisionTree Regressor (CDT) | CausalForestDML(n_estimators=200, min_samples_leaf=10, max_depth=10); CDT(max_depth=6, min_samples_leaf=100) |
| Visualizing ICU risk surface under fentanyl-propofol dose combinations using probability mesh | The dual-exposure cohort | Dose-response surface (probabilistic XGBoost model, 3D mesh) | XGBoost classification model; mesh grid (80x80); z=ICU admission probability; 3D plot |
| Estimating interaction effects of fentanyl and propofol on ICU admission using logistic regression | The adjusted cohort | Logistic Regression with Interaction (statsmodels) | ICU ~ Fentanyl + Propofol + Fentanyl × Propofol; estimated via `statsmodels.logit` |
| Visualizing interaction risk contribution from fentanyl-propofol synergy in ICU admission | The adjusted cohort | Logistic Regression-based Risk Synergy Map | ICU ~ Fentanyl + Propofol + Fentanyl × Propofol; visualization = prob_full - prob_additive |
| Subgroup-specific ICU risk visualization for elderly with normal calcium/platelets and high glucose | The adjusted cohort (Group01: Age≥60, Calcium=N, Platelets=N, Glucose=H) | XGBoost Classifier (Group01 Dose-Response Surface) | n_estimators=300, max_depth=3, learning_rate=0.05, subsample=0.8, colsample_bytree=0.8, reg_alpha=1, reg_lambda=10 |
| Subgroup-specific ICU risk surface for elderly with low hemoglobin, high glucose, and high creatinine | The adjusted cohort (Group02: Age≥60, Hb=L, Glu=H, Cr=H) | XGBoost Classifier (Group02 Dose-Response Surface) | n_estimators=300, max_depth=3, learning_rate=0.05, subsample=0.8, colsample_bytree=0.8, reg_alpha=1, reg_lambda=10 |
| Subgroup-specific ICU risk surface for elderly with high leukocytes, normal urea and hematocrit | The adjusted cohort (Group03: Age≥60, Leukocytes=H, Urea=N, HCT=N) | XGBoost Classifier (Group03 Dose-Response Surface) | n_estimators=300, max_depth=3, learning_rate=0.05, subsample=0.8, colsample_bytree=0.8, reg_alpha=1, reg_lambda=10 |
| Subgroup-specific ICU risk surface for age 45–60 with high urea, low hematocrit, and high leukocytes | The adjusted cohort (Group04: Age 45–60, Urea=H, HCT=L, Leukocytes=H) | XGBoost Classifier (Group04 Dose-Response Surface) | n_estimators=300, max_depth=3, learning_rate=0.05, subsample=0.8, colsample_bytree=0.8, reg_alpha=1, reg_lambda=10 |
| Subgroup-specific ICU risk surface for age 45–60 with high platelets, low calcium, and severe pain | The adjusted cohort (Group05: Age 45–60, Platelets=H, Calcium=L, Pain=Severe) | XGBoost Classifier (Group05 Dose-Response Surface) | n_estimators=300, max_depth=3, learning_rate=0.05, subsample=0.8, colsample_bytree=0.8, reg_alpha=1, reg_lambda=10 |
| Subgroup-specific ICU risk surface for age 45–60 with high creatinine, mild pain, and low hemoglobin | The adjusted cohort (Group06: Age 45–60, Creatinine=H, Pain=Mild, Hb=L) | XGBoost Classifier (Group06 Dose-Response Surface) | n_estimators=300, max_depth=3, learning_rate=0.05, subsample=0.8, colsample_bytree=0.8, reg_alpha=1, reg_lambda=10 |
| Visualize SHAP contribution of Hematocrit under Leukocyte and Urea Nitrogen interactions | The adjusted cohort | XGBoost + SHAP | Default (XGBoost) + SHAP values clipped [-1, 3] |
| Visualize SHAP contribution of Glucose under Calcium and Platelet interactions | The adjusted cohort | XGBoost + SHAP | Default (XGBoost) + SHAP values clipped [-1, 3] |
| Visualize ICU risk from fentanyl and propofol dose interaction in Group04 | The adjusted cohort | XGBoost Classifier | n_estimators=300, max_depth=3, learning_rate=0.05, subsample=0.8, colsample_bytree=0.8, reg_alpha=1, reg_lambda=10, scale_pos_weight=0.5 |
| Visualize ICU risk from fentanyl and propofol dose interaction in Group05 | The adjusted cohort | XGBoost Classifier | Same as Group04 |
| Visualize ICU risk from fentanyl and propofol dose interaction in Group06 | The adjusted cohort | XGBoost Classifier | Same as Group04 |
| To analyze the SHAP interaction effects of hemoglobin on ICU admission risk | The adjusted cohort | XGBoost Classifier | use_label_encoder=False, eval_metric='logloss', random_state=42 |
| To explore the contribution of creatinine influenced by hemoglobin and glucose levels in predicting ICU admission | The adjusted cohort | XGBoost Classifier | use_label_encoder=False, eval_metric='logloss', random_state=42 |
| To investigate the modulation of calcium SHAP value under platelet count and pain score contexts for ICU prediction | The adjusted cohort | XGBoost Classifier | use_label_encoder=False, eval_metric='logloss', random_state=42 |
| **Objective** | **Dataset / Cohort** | **Model** | **Key Parameters** |
| To investigate the modulation of **calcium SHAP value** under **platelet count** and **pain score** contexts for ICU prediction | The adjusted cohort | XGBoost Classifier | use_label_encoder=False, eval_metric='logloss', random_state=42 |
| To estimate **counterfactual ICU risk surface** across joint **fentanyl** and **propofol** doses | The adjusted cohort | XGBoost Classifier | n_estimators=800, max_depth=7, scale_pos_weight, learning_rate=0.1, subsample=0.8, colsample_bytree=0.8 |
| To fit **generalized propensity score (GPS)** models for fentanyl and propofol | The adjusted cohort | XGBoost Regressor | n_estimators=1200, max_depth=6, random_state=42 |
| To compute **residualized dose deviations** for GPS adjustment | The adjusted cohort | XGBoost Regressor | n_estimators=1200, max_depth=6, random_state=42 |
| To explore **ICU admission heterogeneity** by SHAP interaction between **hemoglobin** and **creatinine** | The adjusted cohort | XGBoost Classifier | use_label_encoder=False, eval_metric='logloss', random_state=42 |
| To simulate **counterfactual treatment-response pairs** using joint dose grid | Simulated from adjusted cohort | XGBoost Classifier | Same as above; inference by applying trained model on synthetic GPS-adjusted treatment matrix |

Supplementary Table S11. Logistic regression coefficients and 95% confidence intervals for fentanyl, propofol, and their interaction.

| **Variable** | **Coefficient (β)** | **z-value** | **CI Lower (2.5%)** | **CI Upper (97.5%)** | **Std. Error** | **P** |
| --- | --- | --- | --- | --- | --- | --- |
| Intercept | 0.4300 | 10.604 | 0.351 | 0.509 | 0.041 | < 0.001 |
| Fentanyl | 0.0846 | 4.772 | 0.050 | 0.119 | 0.018 | < 0.001 |
| Propofol | -0.1505 | -8.505 | -0.185 | -0.116 | 0.018 | < 0.001 |
| Interaction | 0.0243 | 3.596 | 0.011 | 0.038 | 0.007 | < 0.001 |


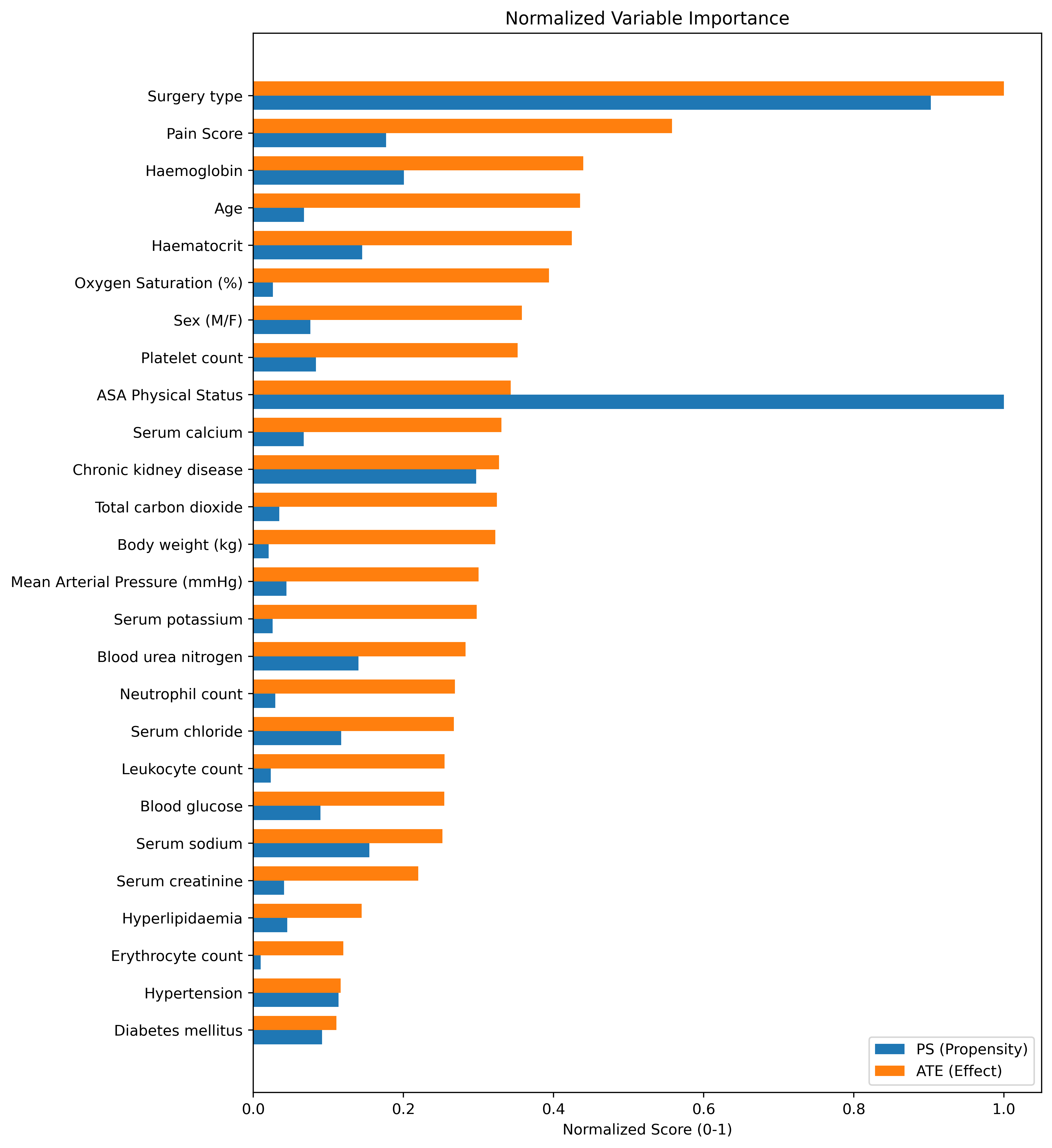


Supplementary Figure S1. Normalized variable importance for propensity score and treatment effect models in total eligible cohort. Variables with high contributions to both PS and ATE were categorized as confounding factors, while the remaining variables were classified as outcome predictors.


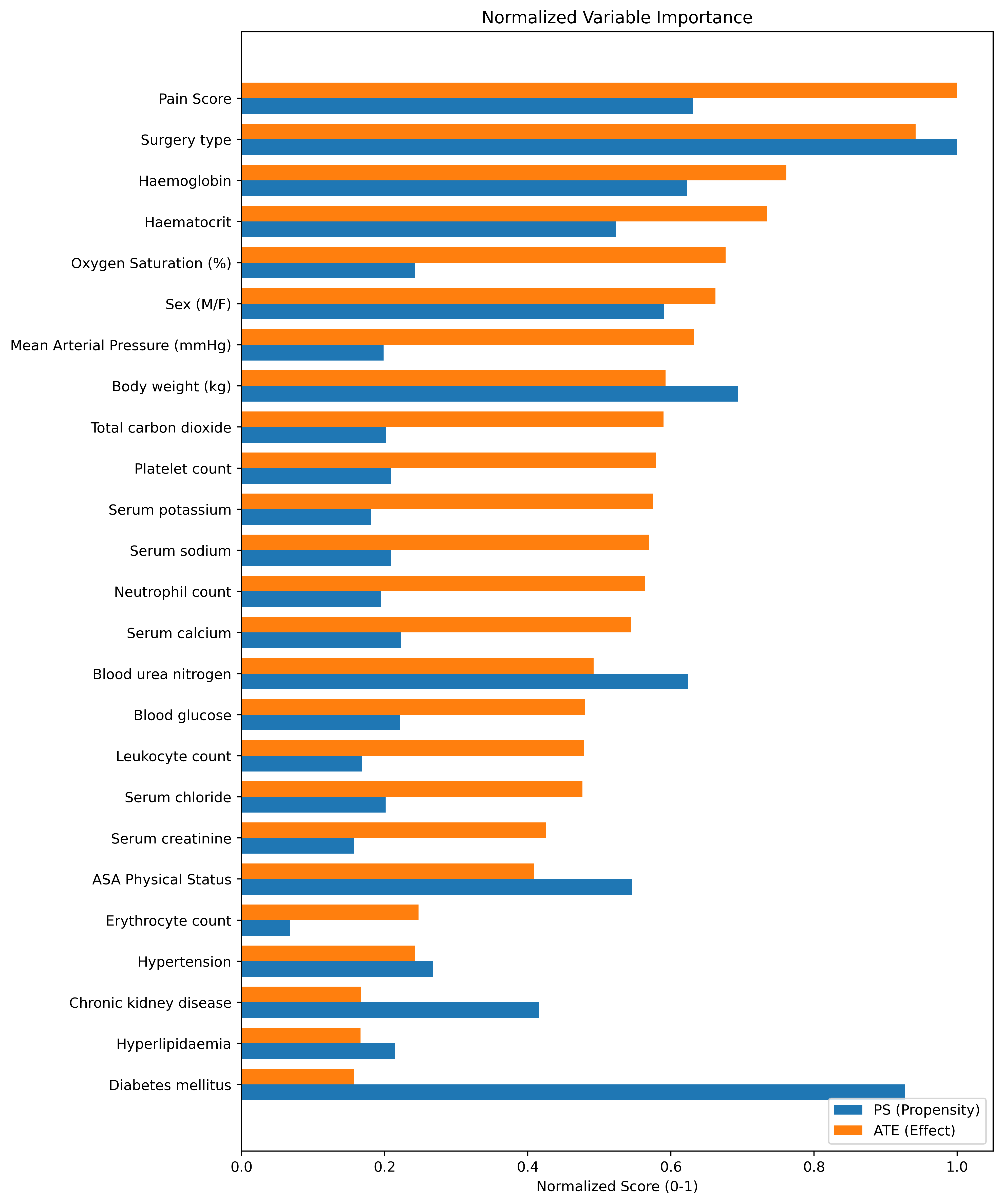


Supplementary Figure S2. Normalized variable importance for propensity score and treatment effect models in dual-exposure cohort. Variables with high contributions to both PS and ATE were categorized as confounding factors, while the remaining variables were classified as outcome predictors. The confounding factors included in the last two cohorts were the same as the outcome predictors.


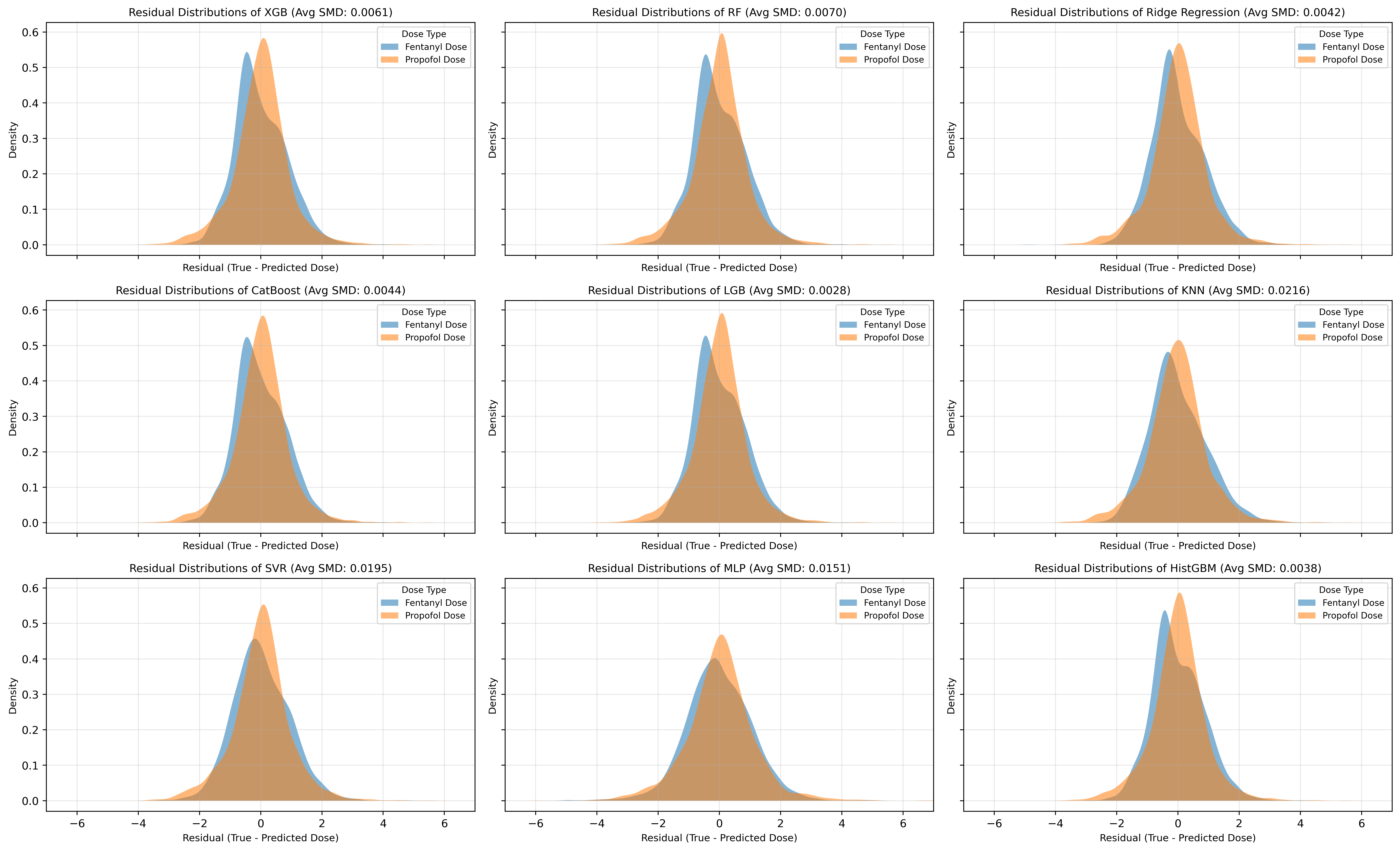


Supplementary Figure S3. Residual distributions of selected covariates before and after IPTW adjustment across nine models. The blue distribution represents residuals from the training set, while the orange distribution represents residuals from the test set. The average standardized mean difference (SMD) between the two distributions is reported in the title of each panel. A lower SMD indicates greater consistency and stability of model performance across training and testing sets, suggesting better generalizability.


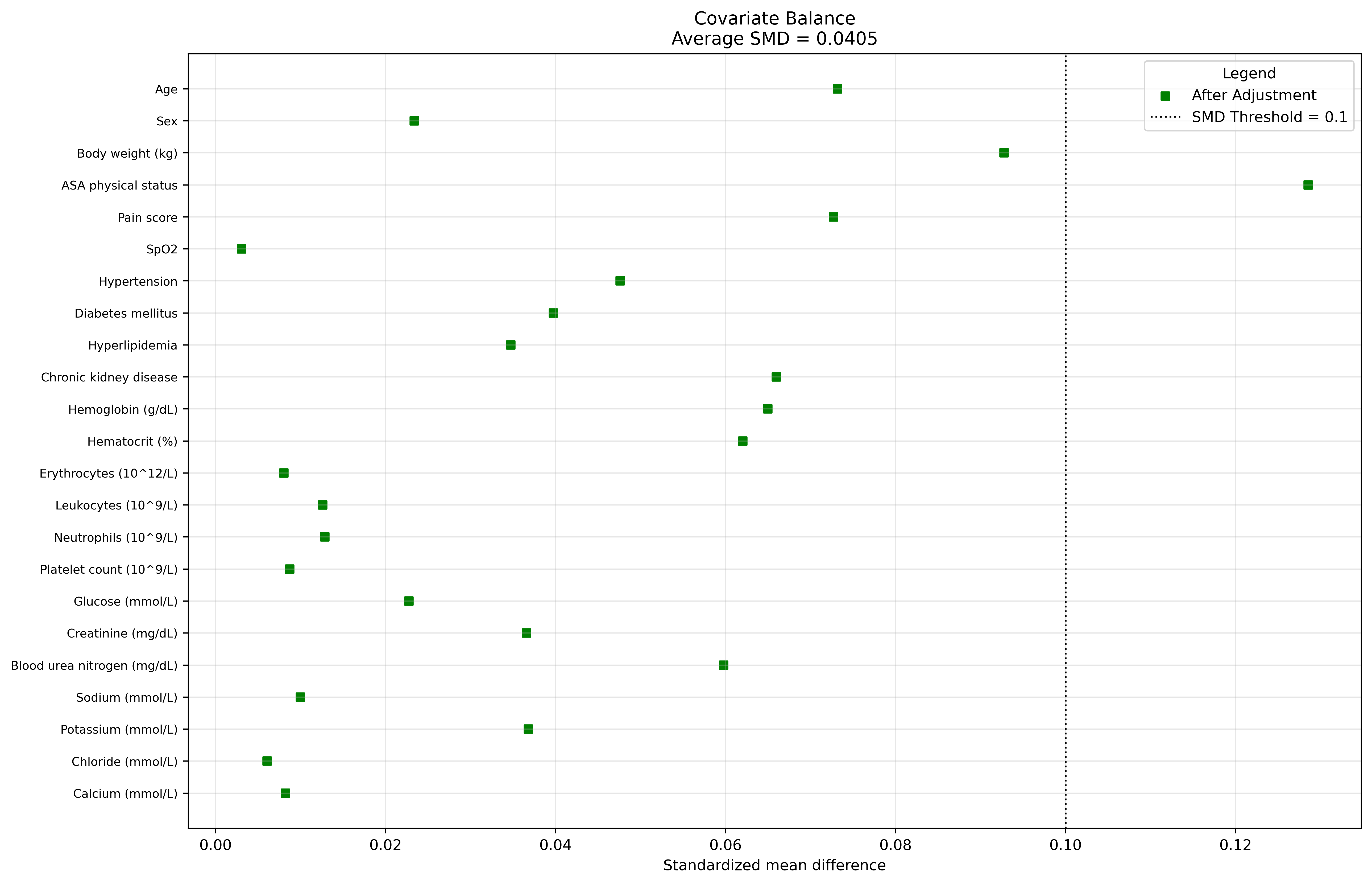


Supplementary Figure S4. Covariate balance after IPTW adjustment. This plot illustrates the SMD of selected covariates after applying inverse probability of treatment weighting. Each green square represents the post-adjustment SMD for an individual covariate. The vertical dotted line at 0.1 denotes the conventional threshold for acceptable covariate balance. Most variables achieved adequate balance (SMD < 0.1), with an overall average SMD of 0.0405. This indicates that the covariates between groups were well-balanced and comparable. Weight was excluded.


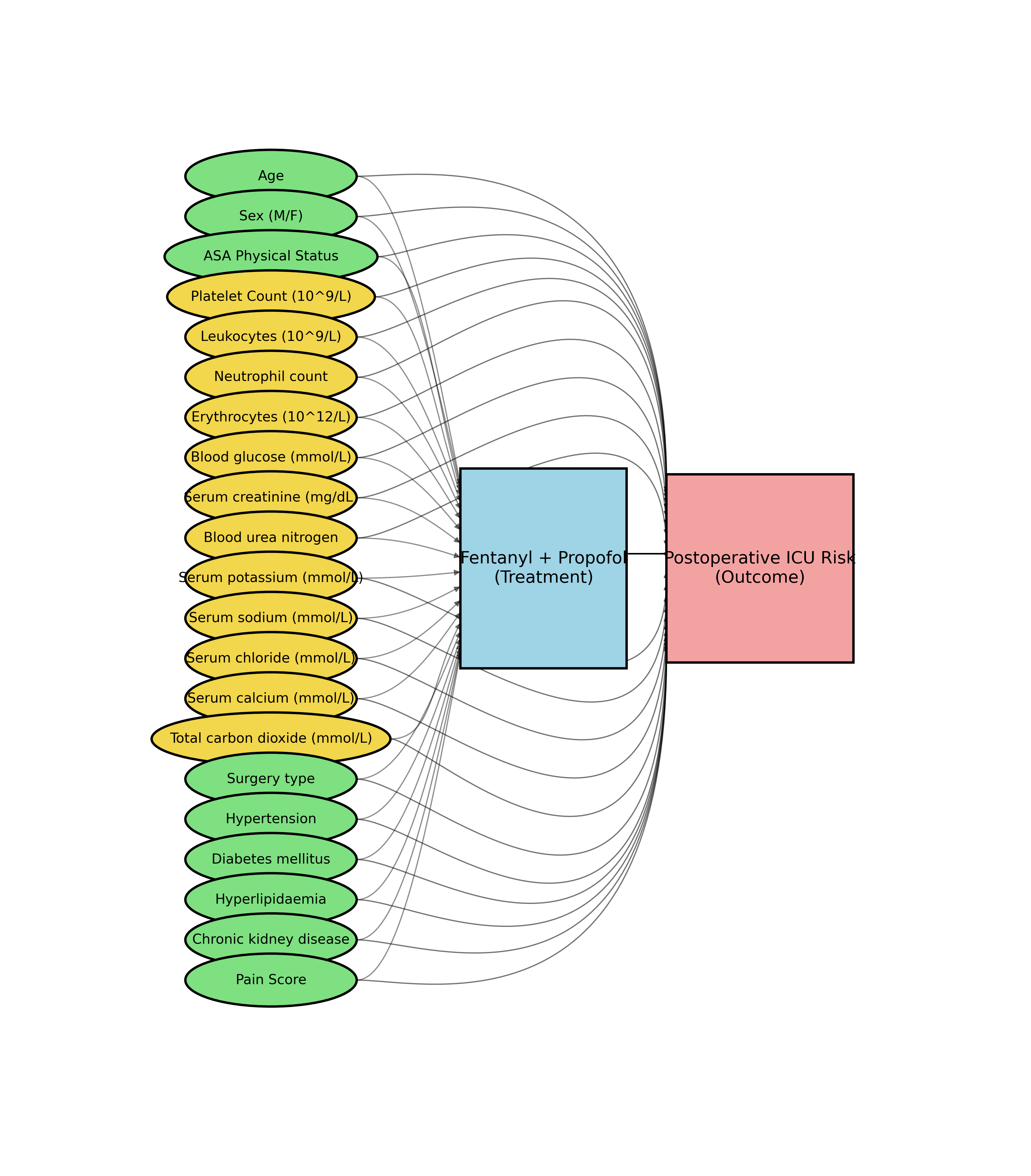


Supplementary Figure S5. Directed acyclic graph of treatment, confounders, and postoperative ICU risk. This directed acyclic graph represents the hypothesized causal structure linking preoperative clinical variables (green: demographics, yellow: laboratory tests, purple: physiological indicators) to the intraoperative co-administration of fentanyl and propofol (blue) and the subsequent risk of postoperative ICU admission (red). Arrows from covariates to both the treatment and outcome indicate potential confounding, implying that these variables may influence anesthetic decision-making as well as postoperative prognosis. The primary causal effect of interest is shown by the arrow from the treatment node to the outcome node, representing the hypothesized impact of drug co-administration on ICU admission risk.


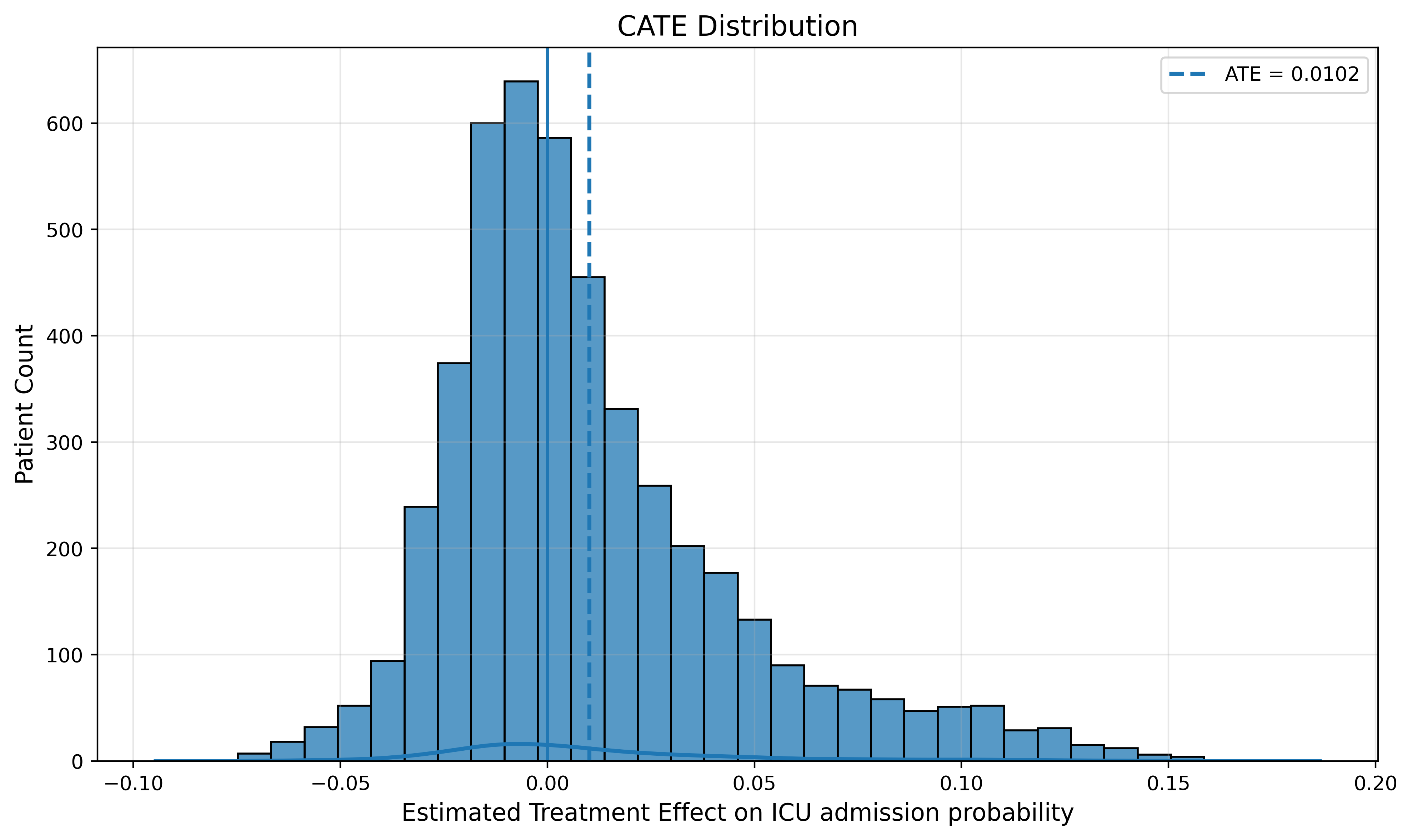


Supplementary Figure S6. Distribution of estimated conditional treatment effects on postoperative ICU admission risk. Histogram of individual-level conditional average treatment effect estimates for ICU admission, derived from a Causal Forest model. The red dashed line indicates the average treatment effect.


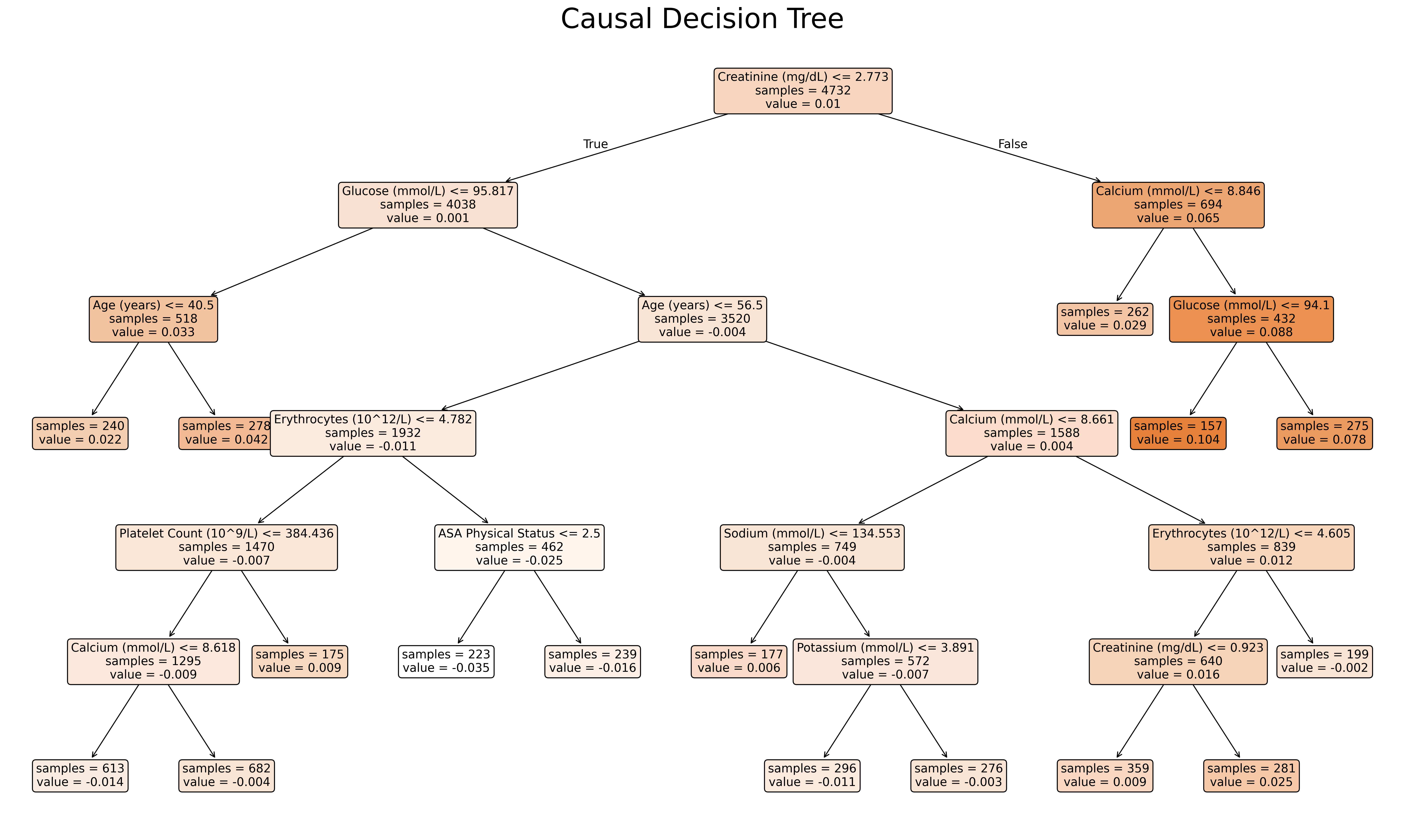


Supplementary Figure S7. Causal decision tree for identifying treatment effect heterogeneity. Each node represents a split on a covariate, and leaf nodes estimate the subgroup-specific conditional treatment effect.





Supplementary Figure S8. Heterogeneous treatment effects across age, platelet count, calcium level, and pain severity. This violin plot illustrates the heterogeneity of estimated conditional average treatment effects (CATE) by age group, stratified across platelet count, calcium level, and pain score subgroups. Each panel corresponds to a unique combination of platelet (Normal, High, Low) and calcium (Low, Normal, High) levels, while the x-axis within each panel represents different age groups (18–44, 45–60, 60+). The distribution of CATE estimates for each pain group (Severe, Mild, Moderate) is shown using color-coded violins. Sample sizes for each group are indicated above the violins.





Supplementary Figure S9. Heterogeneous treatment effects by age group across calcium, platelet count, and glucose level.


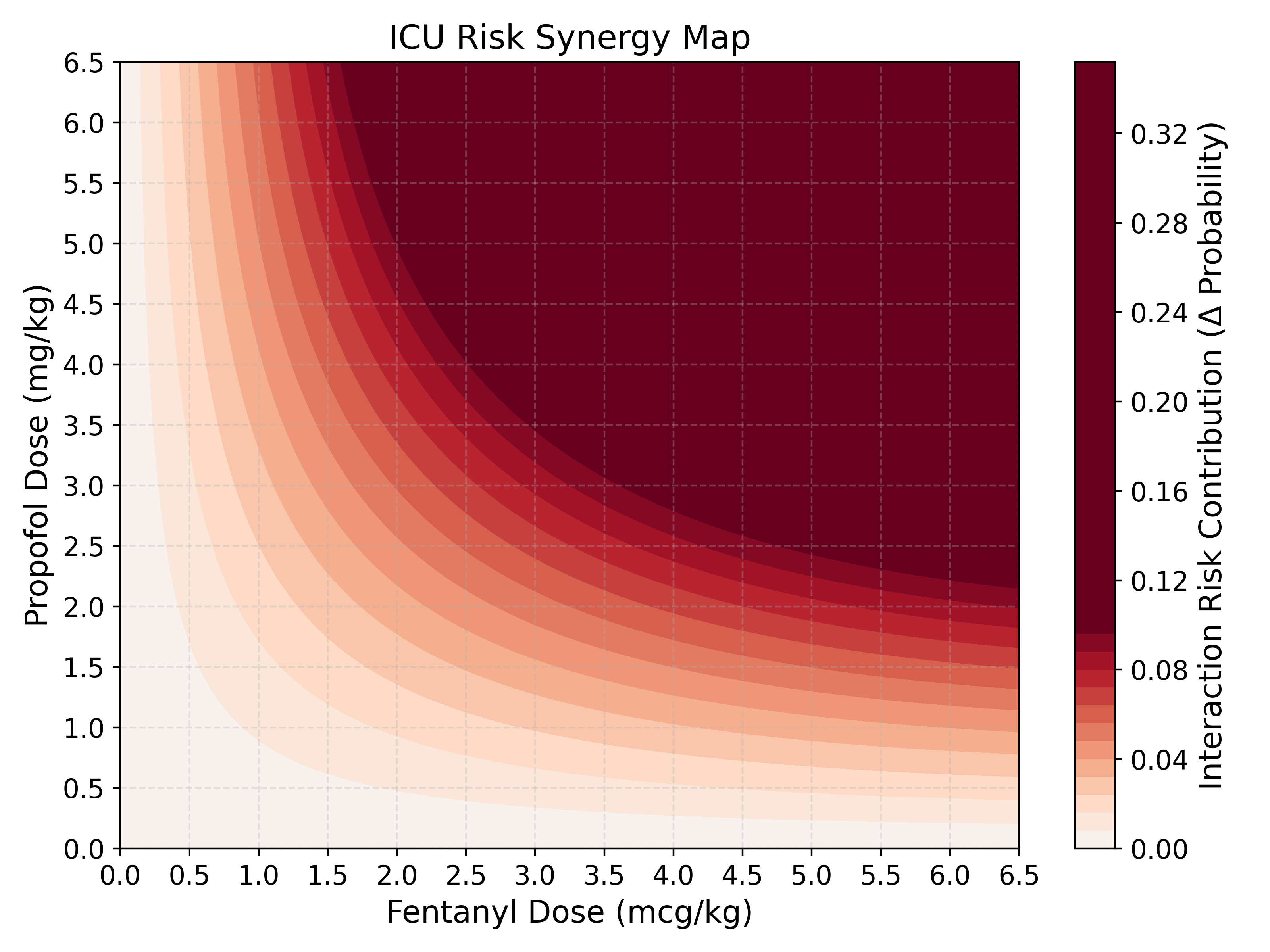


Supplementary Figure S10. Synergy effect plot of fentanyl and propofol on ICU admission risk. We separately calculated the predicted risk surfaces for the model with interaction terms and the model without interaction terms. The difference between two predicted risk surfaces reflected the marginal contribution of the interaction terms to ICU risk.


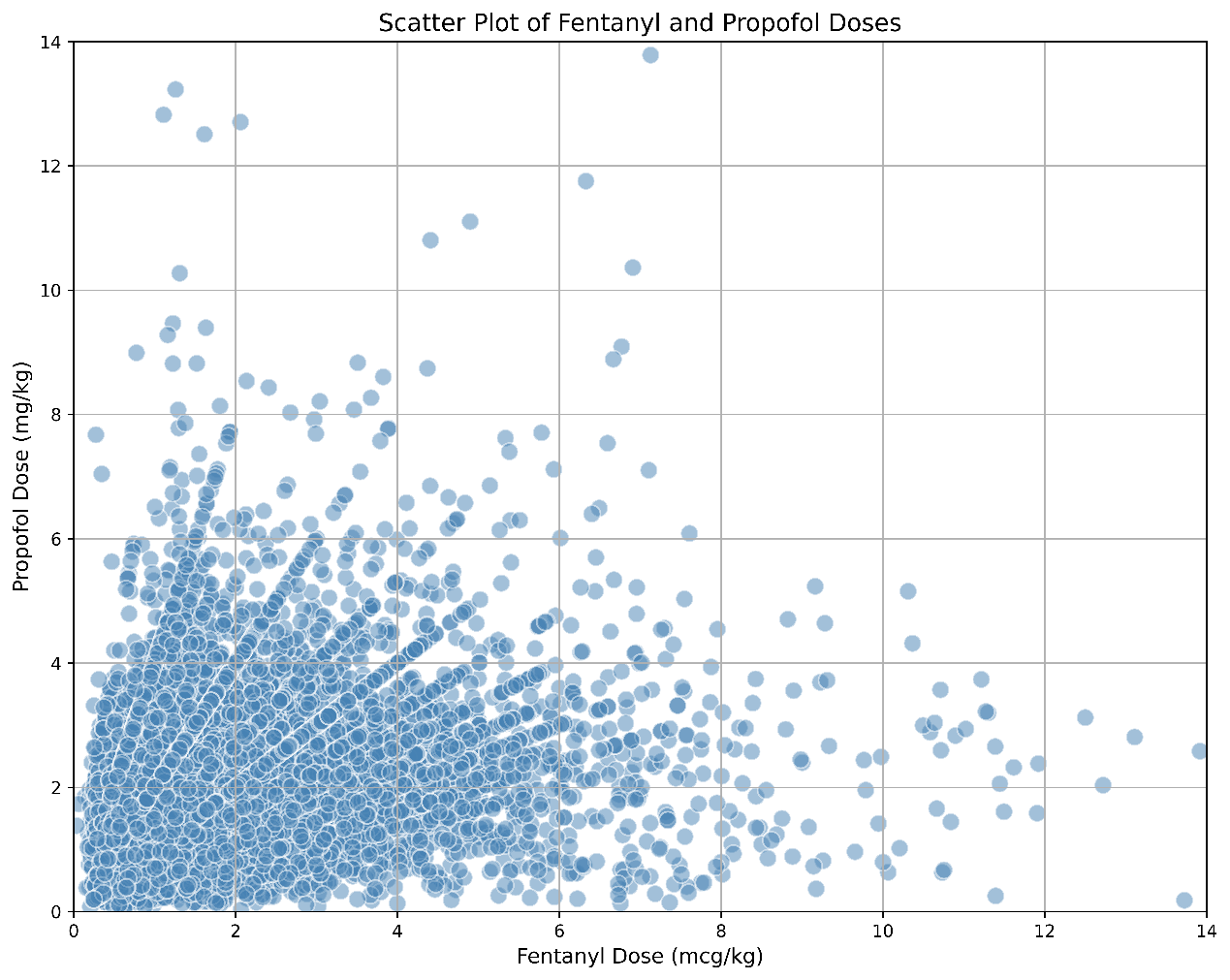


Supplementary Figure S11. Joint distribution of fentanyl and propofol doses. The scatter plot illustrates the empirical joint distribution of fentanyl (mcg/kg) and propofol (mg/kg) doses across all patients included in the analysis. Each dot represents an individual case. The concentration of points in the lower-left quadrant highlights the high-density usage of low to moderate dose combinations, whereas the upper-right region shows markedly fewer samples, reflecting sparse data support in that region.


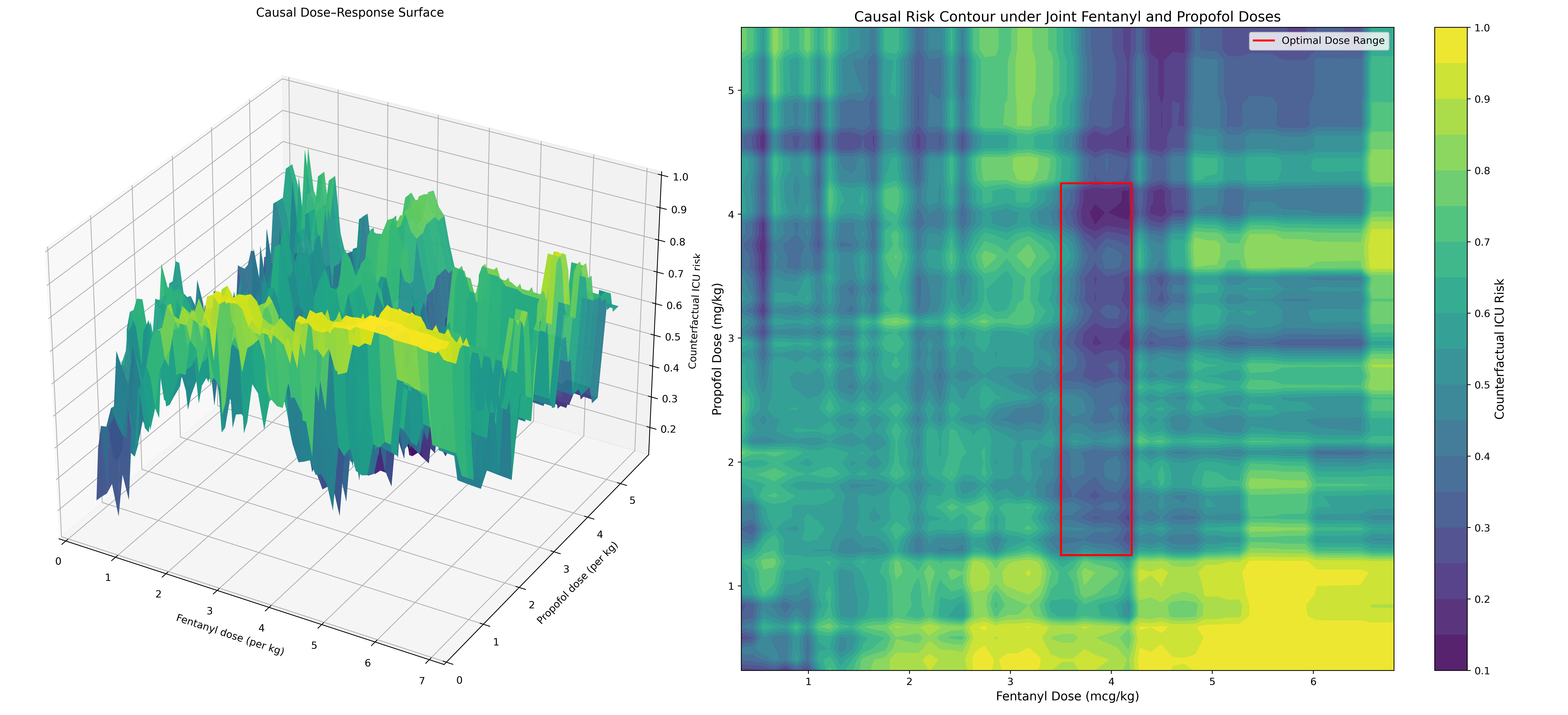


Supplementary Figure S12. Counterfactual risk surface and optimal dose region for joint intraoperative fentanyl and propofol administration. The left panel illustrates the counterfactual dose response surface estimated from a causal machine learning model, representing the predicted postoperative ICU admission risk under varying intraoperative fentanyl and propofol dose combinations. The right panel shows the corresponding contour plot of the counterfactual ICU risk. The red rectangle highlights the empirically identified optimal dose range for decision-making was determined to be 3.5–4.0 mcg/kg for fentanyl and 1.25–4.25 mg/kg for propofol. Within this range, the counterfactual risk is minimized while maintaining clinically effective analgesic and sedative dosing. This region delineates a decision-support zone for individualized and risk-informed dosing strategies under the assumption of conditional exchangeability.
